# Development of a Patient-Specific Finite Element Analysis for Uncemented Total Hip Arthroplasty: A Step Towards Objective Surgical Planning

**DOI:** 10.1007/s10439-026-04033-9

**Published:** 2026-03-13

**Authors:** Vineet Seemala, Mark A Williams, Richard King, Arnab Palit

**Affiliations:** 1 WMG, University of Warwick, Coventry, CV4 7AL UK; 2 Department of Trauma & Orthopaedics, University Hospitals Coventry and Warwickshire NHS Trust, University Hospitals Coventry and Warwickshire, Clifford Bridge Rd, Coventry, CV2 2DX UK

**Keywords:** Finite Element Methods (FEM), Total Hip Arthroplasty (THA), Peri-Prosthetic Fracture (PPF), Digital Volume Correlation (DVC), Surgical planning, Micromotion

## Abstract

**Purpose:**

Aseptic loosening and intraoperative periprosthetic fractures (IOPPFs) are major complications in uncemented total hip arthroplasty (THA). Computational models for assessing primary stability and IOPPF risk preoperatively are limited. This study developed a patient-specific finite element analysis (FEA) framework that replicates stepwise broaching and implantation in uncemented THA, enabling primary stability assessment and providing a foundation for future IOPPF research.

**Methods:**

A FEA framework was developed using patient-specific femoral geometries and heterogeneous material properties to simulate stepwise broaching and implantation along a defined insertion path. Primary stability was assessed via micromotion under physiological loading. Four case studies were performed to (a) demonstrate the framework using three cadaveric femurs, (b) validate the framework against experimental strain measurements obtained via digital volume correlation (DVC), (c) compare predicted outcomes with a literature-based volumetric expansion model, and (d) assess sensitivity to variations in bone material property models.

**Results:**

Average post-implantation von-Mises stress ranged from 9.87 to 14.77 MPa, with each broach increasing stress and indicating progressive bone compaction. Primary stability, assessed via bone-implant micromotion (29.10–78.04 µm), remained well below the 150 µm threshold, considered favourable for osseointegration. The proposed framework showed closer agreement with experimental DVC strains and compared to the volumetric-expansion model, halved the prediction error. The analysis also demonstrated limited sensitivity to variations in *E-ρ* models.

**Conclusion:**

The proposed FEA method replicates stepwise broaching and implantation in uncemented THA, enabling patient-specific assessment of bone-implant interactions and primary stability, and providing a foundation for preoperative tools to evaluate IOPPF and aseptic loosening risk and guide tailored femoral implant selection.

**Supplementary Information:**

The online version contains supplementary material available at https://doi.org/10.1007/s10439-026-04033-9.

## Introduction

Total Hip Arthroplasty (THA) effectively relieves pain and restores mobility in hip osteoarthritis. Implants may be cemented using polymethyl methacrylate (PMMA) or uncemented, relying on press-fit fixation for primary stability, followed by osseointegration [1–3]. Although modern cemented implants perform well, concerns such as low tensile strength and osteolysis have shifted preference towards uncemented designs [4, 5]. In the UK (excluding Scotland), the use of cemented implant declined to one-third of its 2004 level, while uncemented use rose 2.6-fold [6]. By 2030, THA procedures in young adults are projected to increase fivefold, with over 80% involving uncemented implants [6, 7].

Intraoperative periprosthetic fractures (IOPPF) are a major concern in uncemented THA, linked to a 7.2-fold increased risk of aseptic loosening, greater post-revision complications, and higher postoperative periprosthetic fractures (POPPF) incidence [8]. PPF frequency is projected to rise by 4.6% per decade over the next 30 years [9], with revision rates due to PPF increasing from 13.1 to 20.4% in the past 5 years [6]. Implant type strongly influences complication risk, with uncemented implants, where IOPPF typically occurs during broaching and implantation, being over three times more likely to cause IOPPF than cemented implants [10–12]. Predicting IOPPF remains challenging due to patient-specific bone geometry and morphology [13–15].

Aseptic loosening, the leading cause of revision surgery in the UK (excluding Scotland), accounts for 45.4% of all revision cases [6] and is closely linked to increased micromotion at the bone–implant interface resulting from insufficient primary implant stability. Excessive micromotion impedes osseointegration of uncemented implants, compromising long-term stability. However, assessing implant stability in terms of micromotion remains difficult. Together, aseptic loosening and PPFs account for nearly two-thirds of all revision surgeries [6]. Despite these challenges, current surgical planning for implant selection and positioning primarily considers dimensional fit within the bone anatomy, limiting the ability to predict IOPPF risk or evaluate primary implant stability based on micromotion.

Finite Element Analysis (FEA) is a powerful tool for investigating bone–implant interactions and uncemented THA performance [16–23]. Although FEA has been applied to assess postoperative stability in terms of micromotion at the bone-implant interface and POPPF risk [16, 20], its use in evaluating IOPPF remains limited [22], despite IOPPFs occurring more frequently than POPPFs [14]. Current models often oversimplify stepwise broaching and implantation process, simulating them as volumetric expansion [17] or opposing forces [18, 19], thus failing to capture the complex bone-broach and bone-implant interactions throughout their insertion path during the uncemented THA. This oversimplification can compromise the accuracy of stress-strain predictions, their distributions, and subsequent assessments of IOPPF risk and implant stability based on micromotion. To the authors’ knowledge, no studies have employed FEA to simulate the complete stepwise broaching and implantation process, despite its critical role in primary stability and IOPPF risk assessment.

Therefore, the aim of this study was to develop an FEA framework that replicates the femoral broaching and implantation steps involved in uncemented THA, establishing a foundation for preoperative planning tools to evaluate IOPPF and aseptic loosening risk and to support tailored femoral implant selection for improved surgical outcomes.

To achieve this, the study pursued the following objectives:

A. To define broach and implant paths within the femoral canal as intended in uncemented THA.
B. To construct an FEA framework capable of replicating the femoral stepwise broaching and implantation, following the paths defined in first objective, and to assess the primary stability of the implant under loading conditions representative of activities of daily living (ADLs).
C. To demonstrate the novelty of the proposed model by conducting a cadaveric case study, validating the simulation results against experimental data, and comparing them with the recent *Volumetric Expansion Method* developed by Esposito et al. [17], which uses a simplified volume-expansion approach for modeling uncemented THA.

## Methods and materials

**Fig. 1:**
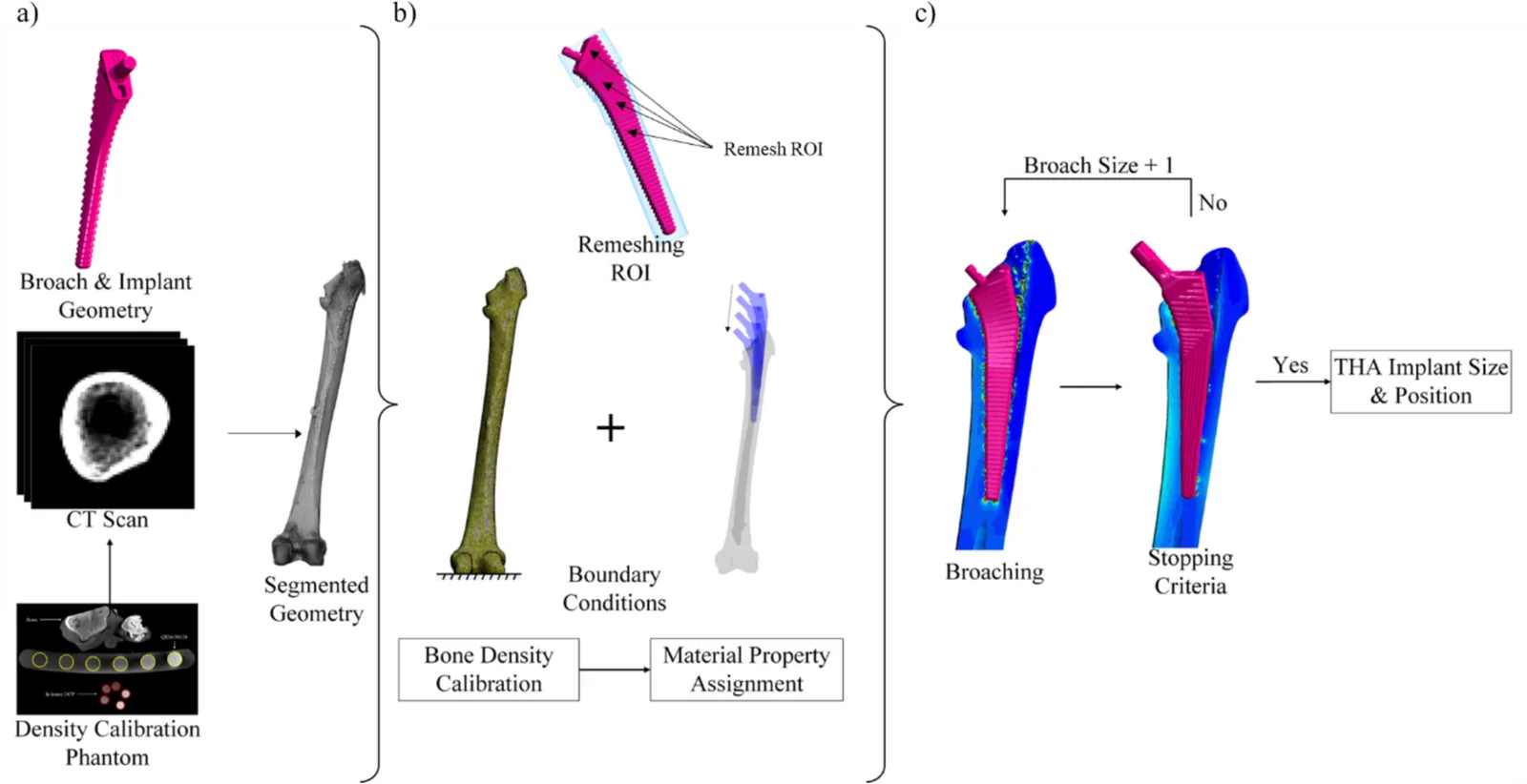
FEA Workflow**. a** Inputs required for the FEA, including CT imaging of the patient’s femur, bone density distribution, and the geometries of the broach and implant; **b** FEA setup, involving the specification of material properties, definition of boundary conditions, and designation of the local remeshing ROI; **c** FEA simulation of the broaching and implantation procedures, conducted until the predefined stopping criteria are satisfied

This section outlines the development of a novel FEA framework (*Proposed FEA Method*) that replicates the stepwise broaching and femoral implantation process in uncemented THA (Fig. 1), including an assessment of primary implant stability. Subsequently, four case studies were conducted to demonstrate the capability of the proposed method, validate the framework against cadaveric experiments, compare it with the volume-expansion-based FEA model [17] (*Volumetric Expansion Method*), and evaluate its sensitivity to bone material properties.

### Development of novel FEA simulation

#### Broaches, implants, and patient-specific femur geometry

Implant and broach geometries were obtained from CAD models or reconstructed via laser scanning (Fig. 1a). Surface meshes derived from laser scans were remeshed to generate closed, artifact-free surfaces with a maximum edge length of 1 mm to prevent stress concentration and convergence issues during FEA simulations. No modifications were made to the CAD models obtained directly from the manufacturer. Patient-specific femoral geometry was extracted from medical-CT scans, and density calibration was performed by converting CT scan intensities to bone density using a density calibration phantom [24] (Fig. 1a). Femurs were segmented using watershed methods in Avizo 3D 2021.2 (Thermo Fisher Scientific, Germany) and meshed with 10-node tetrahedral elements. The element size was determined through a mesh convergence study, with a maximum element size of 2 mm (Fig. 9 in Appendix A). A femur-specific local coordinate system was defined using femoral landmarks identified from CT data, following ISB guidelines [25–27]. Femurs, with their femur-specific local coordinate frames defined, were aligned to the global reference frame representing the anatomical reference frame to enable comparability across scans and geometries along the anatomical directions, thereby enhancing clinical relevance. An *in-silico* neck osteotomy was performed at the piriformis insertion following surgical standards.

#### Broach and implant path definition

**Fig. 2:**
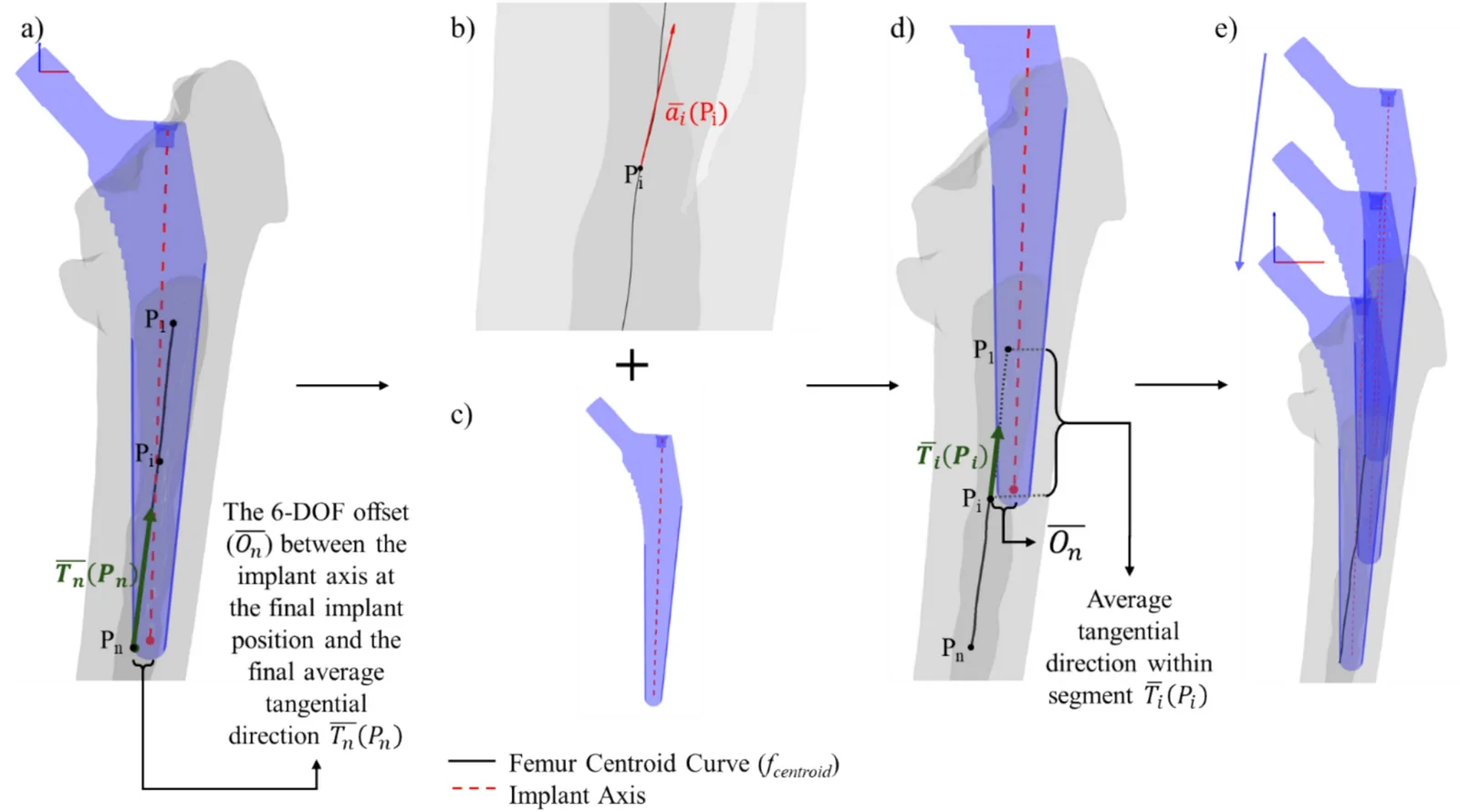
Broach/Implant Path Definition: **a** Final implant position in segmented femur geometry with the centroid curve highlighted as a solid black line defined between points *P*_*1*_ and *P*_*n*_. The 6-DOF offset $\overline{{O }_{n}}$ is calculated between the implant axis at the final position (measured using standard dimensional methods) and the final average tangential direction $\overline{{T }_{n}}\left({P}_{n}\right)$; **b** Tangential direction of the centroid curve ($\overline{{a }_{i}}\left({P}_{i}\right)$) at point *P*_*i*_, indicated by the red direction vector; **c** Broach/implant axis defined according to surgical guidelines, shown as a dotted red line; **d** Broach/implant orientation determined using the average tangential directions $\overline{{T }_{i}}\left({P}_{i}\right)$ of the centroid curve and the implant axis at point *P*_*i*_, as described in Equation (2) while maintaining the 6-DOF offset $\overline{{O }_{n}}$; **e** Broach/Implant path into the femoral cavity

A critical and novel aspect of this study is the definition of the broach or implant insertion path based on the final implant position, which is determined by the surgeon using patient-specific bone geometry, implant size, and established surgical guidelines (Figs. 1b and 2e). The final implant position serves as the reference for calculating the rigid-body six-degree of freedom (6-DOF) offset ($\overline{{O }_{n}}$), between the implant axis at this final position and the corresponding average tangential direction of the femoral centroid curve ($\overline{{T }_{n}}\left({P}_{n}\right)$) (Fig. 2a and Equation (2)). Additionally, the depth of implant insertion from the proximal end of the femur is determined at this stage. Following this, all broach sizes up to the size corresponding to the final implant were aligned to the final implant position using the same proximal features that interface with the broach handle (Appendix B), while preserving $\overline{O}_{n}$, in order to compute the insertion depth for each broach.

A centroid curve (*f*_*centroid*_) was generated based on the segmented surface of the femoral canal, extending from the proximal end of the canal (*P*_*1*_) to the final depth of implant insertion (*P*_*n*_). This curve was discretised into *n* segments (typically 25-30 points, depending on femur size). At each discrete point *P*_*i*_ along the curve (*i* ∈ [1, *n*]), the local tangential direction $\overline{{a }_{i}}\left({P}_{i}\right)$ was computed (Fig. 2b and Eq. (1)):

1 $$\overline{{a }_{i}}\left({P}_{i}\right)= \overline{\nabla }{f }_{centroid}\left({P}_{i}\right)$$

The central axes of the broach and implant were defined from the impaction handle insertion to the distal tip of the implant (Fig. 2c). The insertion path was constructed by aligning the implant axis to remain parallel to the average tangential direction ($\overline{{T }_{i}}\left({P}_{i}\right)$) and by maintaining $\overline{O}_{n}$, starting from the proximal end *P*_*1*_ to the insertion point *P*_*i*_ (Fig. 2d). The average tangential direction at each step *i* is defined by Eq. (2), where *k* is a dummy index ranging from *1* to *i*:

2 $$\overline{{T }_{i}}\left({P}_{i}\right)=\frac{\left(\frac{{\sum }_{k=1}^{i}\overline{{a }_{k}}}{i}\right)}{\left|\frac{{\sum }_{k=1}^{i}\overline{{a }_{k}}}{i}\right|} i\in \left[1,n\right]$$

At each point *P*_*i*_. the implant axis orientation was defined by applying the $\overline{O}_{n}$ to the average tangential direction $\overline{{T }_{i}}\left({P}_{i}\right)$ (Fig. 2d). The complete 6-DOF pose of the implant was computed relative to the initial position (*P*_*1*_) for each broach size and for the final implant (Fig. 2e). All computations were performed using MATLAB R2022b (The MathWorks, Natick, MA, USA).

To mitigate edge effects near the proximal entry of the canal, caused by insufficient surface mesh density resulting in excessive curvature and unreliable tangential direction estimates, a positional offset was introduced. This offset also served to simulate the mechanical hammering effects observed during broaching and implantation in cadaveric experiments. The prescribed displacements and Euler angle rotations of the broach/implant along its defined path into the femur, relative to the initial implant position, are provided in Fig. 11 of Appendix B.

#### Material property definition

In this study, bone was modelled as a heterogeneous, isotropic bilinear material, with Young’s modulus [28] and yield stress [29, 30] defined as functions of bone density (Eqs. 3 and 4). After the yield stress was reached, the tangent modulus was set to 5% of the initial Young’s modulus [31, 32]. Density was obtained by calibrating CT intensities and assigned voxel-wise [24]. A maximum principal plastic strain of 0.01 [33] was employed as the failure criterion to simulate trabecular strut breakage due to compaction during broaching and implantation, as well as cortical fractures leading to PPF. Element deactivation in Marc Mentat 2023.3 (MSC Software Corporation, Hexagon, Sweden) was used to simulate progressive fracture by removing elements that exceeded this threshold. Apparent density was converted to ash density via Eq. (5):

3 $$E=6580{ \rho }_{app}^{1.49}$$

4 $${\sigma }_{ys}=\left\{\begin{array}{c}137 {\left({\rho }_{ash}\right)}^{1.88} ~~~ for ~~~ {\rho }_{ash}<0.317 g/cc\\ 114{ \left({\rho }_{ash}\right)}^{1.72} ~~~ for ~~~ {\rho }_{ash}\ge 0.317 g/cc\end{array}\right.$$

5 $${\rho }_{ash}=0.55{ \rho }_{app}$$

Young’s modulus at each mesh integration point was assigned by mapping the nearest voxel using the *‘hooklw’* subroutine. Plastic behaviour and failure criteria were implemented via the *‘wkslp,’ ‘ufail,’* and *‘uactive’* subroutines in Marc Mentat 2023.3 (MSC Software Corporation, Hexagon, Sweden). Voxel locations from the CT scans were determined using in-house MATLAB code (Fig. 1b).

#### Boundary and loading condition

Boundary conditions replicated intraoperative constraints by fully fixing the femur’s distal end to prevent translation and rotation, simulating leg stabilisation during surgery (Fig. 1b). Prescribed displacements and rotations were applied to the broach or implant to follow the path described in Sect. “Broach and implant path definition”, representing advancement of broaches/implant into the femoral canal driven by repeated mallet strikes during broaching and implantation. The initial and final positions and poses of all broach sizes and the implant are identical for a given femur sample, therefore, the defined path remains the same across all broach sizes and the implant for a given femur sample. The calculation procedure for the prescribed displacements and rotations for each broaching and the implantation step is described in detail in Appendix B.

#### Broaching and implantation simulation

A frictional contact coefficient of 0.5 was assumed between broach/implant and bone [34–36]. The broach and implant were modelled as rigid bodies, as their metallic stiffness is several orders of magnitude greater than that of bone [17, 37–40]. To improve computational efficiency compared with global mesh refinement, and to enable the simulation of local bone failure through material removal via element deactivation, a two-level local adaptive remeshing strategy was implemented within four cylindrical regions surrounding the broach or implant (Fig. 1c). These regions were defined by the broach or implant geometry with an additional 2 mm padding, while the mesh size outside these zones was maintained according to the mesh convergence criteria (Appendix A). This local refinement allowed targeted element deactivation by removing smaller regions after failure, preserving the overall bone–implant contact and reducing computational cost compared with global uniform remeshing. The 2 mm padding area was selected based on the depth of bone compaction reported in previous studies [41, 42]. Remeshing within the ROI was chosen over failure-based remeshing after initial tests with various local remeshing criteria, including *nodes on contact*, *equivalent strain*, *equivalent plastic strain*, and *nodes within a defined region*, showed that ROI remeshing more accurately captured trabecular bone compaction.

Simulations progressed sequentially from the smallest broach size (0) to the final implant, following the surgeon’s planned size and position based on only dimensional information. The initial simulation used the undeformed femur with neck cut from preoperative-CT. Subsequent steps used the remeshed, deformed geometry from the prior simulation to maintain mesh quality as per the convergence study (Fig. 9 in Appendix A). For example, after broach size 1 simulation, the deformed femur was remeshed and used as input for broach size 2.

To address stem positioning uncertainty between the planned and actual implant placement, the implant path was perturbed proximodistally within 0.1 ± 2.3 mm [43, 44] from the planned location following the final implantation simulation. The FEA results were evaluated at each incremental location between the nominal (planned) implant position and the extreme position in the proximodistal direction to assess the impact of implant positioning uncertainty. This approach provides the expected range of FEA results for each location that could occur for a given implant positioning, accounting for the discrepancy between the planned and achieved implant placement during surgery.

#### Assessment of primary stability

Post-implantation primary stability was assessed by applying a load of 251% of body weight (BW) at the hip joint centre, simulating the maximum load experienced during ADL [45]. The loading directions were defined by the average force angles, *α*_*front*_ and *α*_*sagit*_ relative to the anatomical planes, corresponding to the most frequent (walking) and the most demanding (stair ascent/descent) activities, with values of 17.5° and 13.5°, respectively, based on previously reported hip joint contact forces and suggested standard for evaluating primary stability of THA measured using instrumented implants [45, 46]. The load was applied at the hip joint centre with the distal femur fixed in all six-DOF, and no muscle-reaction forces were considered, consistent with prior studies [46]. The load was linearly increased from 0 to 251% of body weight.

Micromotion at the bone-implant interface, defined as the relative movement between bone and implant, was quantified using the ‘*Accumulated Contact Slip*’ parameter in Marc Mentat 2023.3 (MSC Software Corporation, Hexagon, Sweden). The ‘*Accumulated Contact Slip*’ represents the relative tangential displacement between the two contact bodies at the interface, calculated from the nodal displacement values of the bone and implant, and accumulated over the duration of the load case. The average micromotion was then compared against an established threshold for average micromotion of 150 µm [47–50] to evaluate primary stability in terms of allowable micromotion that promotes osseointegration and ensures secondary stability of the implant. All assessments were conducted at the nominal (planned) implant position, and micromotion was specifically quantified at the bone-implant interface.

In the proposed FEA framework, implant stability can be assessed using two distinct approaches:

1. **Without preoperative planning data**: Stability is assessed incrementally by inserting implants corresponding to each broach size increase. Micromotion is evaluated at each step, with broach size increased until the osseointegration threshold is satisfied.
2. **With preoperative planning data:** Stability is directly assessed using the planned implant size and position, improving computational efficiency. If stability is insufficient, alternative sizes can be evaluated to ensure osseointegration. In the following case studies, this approach was applied to evaluate primary stability, as both the preoperative planning data and the achieved implant position were available, as detailed in the subsequent section.

### Case studies

#### Study 1: Demonstration of the proposed FEA framework using a cadaver experiment

Three cadaveric femur samples (age-sex-side: 70-male-left, 76-female-right, 78-male-right) were used in this study. Ethical approval was granted by the University of Warwick Biomedical and Scientific Research Ethics Committee (Ref: 66/22-23) and UHCW NHS Trust R&D Department (Ref: GF0503). THA procedures were performed by an orthopaedic surgeon with over 25 years of experience. Medical-CT scans were acquired using a GE Revolution CT scanner (120 kV, voxel size 0.5 × 0.5 × 0.625 mm). Metafix broaches and implants (Corin, UK) were laser scanned with a Nikon ModelMaker-H120 (WMG). Further experimental details are provided in Seemala et al. [24, 42]. Implant sizes 8, 6, and 4 were used for femurs 1, 2, and 3, respectively, following surgical planning guidelines.

The FEA framework replicated stepwise broaching and implantation for each femur sample as follows. Femur sample 1 involved 10 steps, progressing from broach size 0 to size 8, followed by the implantation step. Femur sample 2 included 8 steps, from broach size 0 to size 6, also followed by an implantation step. For femur sample 3, the process consisted of 6 steps, from broach size 0 to size 4, with a final implantation step. The prescribed displacements and rotations applied to the broach or implant, along with their defined paths, are provided in Fig. 10 of Appendix B for all three femur samples.

The stress and strain distributions during bone-broach and bone-implant interactions were quantified using von Mises stress (*σ*_*VM*_) and equivalent microstrain (*με*_*eq*_) along the implant axis direction, from proximal to distal, within a 5 mm region from the implant surface around the bone-implant interface with the implant in the nominal (planned) location. The FEA simulations were based on the measured final implant positions achieved during the surgical procedures to accurately reflect implant placement in the computational model.

#### Study 2: Validation of the proposed FEA using a cadaver experiment-based strain calculation

The FEA model proposed in this study was validated by comparing the predicted femoral strains post-implantation to reference strains measured via Digital Volume Correlation (DVC) from high-resolution micro-CT scans, as reported in Seemala et al. [42]. The comparison accounted for implant positioning uncertainty between the planned and achieved implant locations, as described in Sect. “Broaching and implantation simulation”, as well as the measurement uncertainty inherent to DVC measurement. Strain assessments focussed on equivalent microstrain (*με*_*eq*_) along the implant axis direction from proximal to distal. At each location, the strain within a 5 mm region from the implant surface around the bone-implant interface was calculated by identifying the closest corresponding node and its nodal value between the DVC and FEA datasets.

#### Study 3: Comparison with the literature

The recent FEA model by Esposito et al. [17] (*Volumetric Expansion Method*), simulating uncemented THA, was reproduced for comparison using the final implant position from the case study (Sect. “Broaches, implants and patient-specific femur geometry”). This model simulates implantation through a uniform 12.5% volumetric expansion of the implant to approximate cumulative mechanical loading. However, it does not explicitly model sequential surgical steps, such as the insertion process and stepwise broach size increments, necessary for capturing detailed bone-broach and bone-implant interactions. Furthermore, the uniform 12.5% expansion may not be appropriate across different implant sizes and does not account for size-dependent variations. The heterogeneous material model definition used by Esposito et al. [17] was adopted in the reproduction.

To compare methodologies, two FEA simulations were conducted for each femur specimen: the proposed framework and the approach developed by Esposito et al. [17] (*Volumetric Expansion Method*). Simulation outcomes were evaluated against experimentally measured strains from DVC by comparing equivalent microstrain (*με*_*eq*_) along the implant axis direction, from proximal to distal, within a 5 mm region from the implant surface around in the bone-implant interface, as well as stress distributions within the femoral geometry. The mean difference between the strain values obtained from the two FEA methods: the ‘*Proposed FEA Method*’ and the ‘*Volume Expansion Method*’, relative to the DVC strain values was calculated at each location by identifying the closest corresponding node to each DVC point and using its associated nodal value from both FEA methods.

#### Study 4: Bone material property sensitivity on FEA

Sensitivity of the elastic modulus-density (*E-ρ*) relationship was assessed using eight *E-ρ* models summarised in Table 1, while plasticity and failure criteria were kept same. The analysis used the implantation FEA simulation of femur sample 1 from the cadaver study. Root means square error (RMSE) quantified differences between FEA-predicted strains for each model and experimentally measured DVC strains.

**Table 1:** Eight elastic modulus–density (*E-ρ*) relationships used for sensitivity analysis with consistent plastic region correlations and failure criteria

Mat	Correlation	References
Elastic region		
1	$E=6850{\rho }_{app}^{1.49}$	[28]
2	$E=10095{\rho }_{ash}$	[51]
3	$E=12000{\rho }_{ash}^{1.45}$	[52]
4	$$\begin{array}{c}E=33900{\rho }_{ash}^{2.20} ~~~ for ~~~ {\rho }_{ash}\le 0.27\\ E=5307{\rho }_{ash}+469 ~~~ for ~~~ 0.27<{\rho }_{ash}<0.60\\ E=10200{\rho }_{ash}^{2.01} ~~~ for ~~~ 0.6\le {\rho }_{ash}\end{array}$$	[29, 30]
5	$E=10200{\rho }_{ash}^{2.01}$	[30]
6	$E=15100{\rho }_{QCT}^{2.25}$	[53]
7	$E=10500{\rho }_{ash}^{2.29}$	[30]
8	$$\begin{array}{c}E=3162{\rho }_{app}^{2.5} ~~~ for ~~~ {\rho }_{app}\le 1.2\\ E=2733{\rho }_{app}^{3.3} ~~~ for ~~~ {\rho }_{app}>1.2\end{array}$$	[54]
$${\rho }_{app}={\rho }_{ash}/0.55$$		[29]

*Plastic region*		
$$\begin{array}{c}{\sigma }_{Y}=137{\rho }_{ash}^{1.88} ~~~ for ~~~ {\rho }_{ash}<0.317\\ {\sigma }_{Y}=114{\rho }_{ash}^{1.72} ~~~ for ~~~ {\rho }_{ash}\ge 0.317\end{array}$$		[29]

*Failure criteria*		
$${\epsilon }_{plasic}\ge 0.01$$		[33]

## Results

### Study 1 results: Demonstration of the proposed FEA

**Fig. 3:**
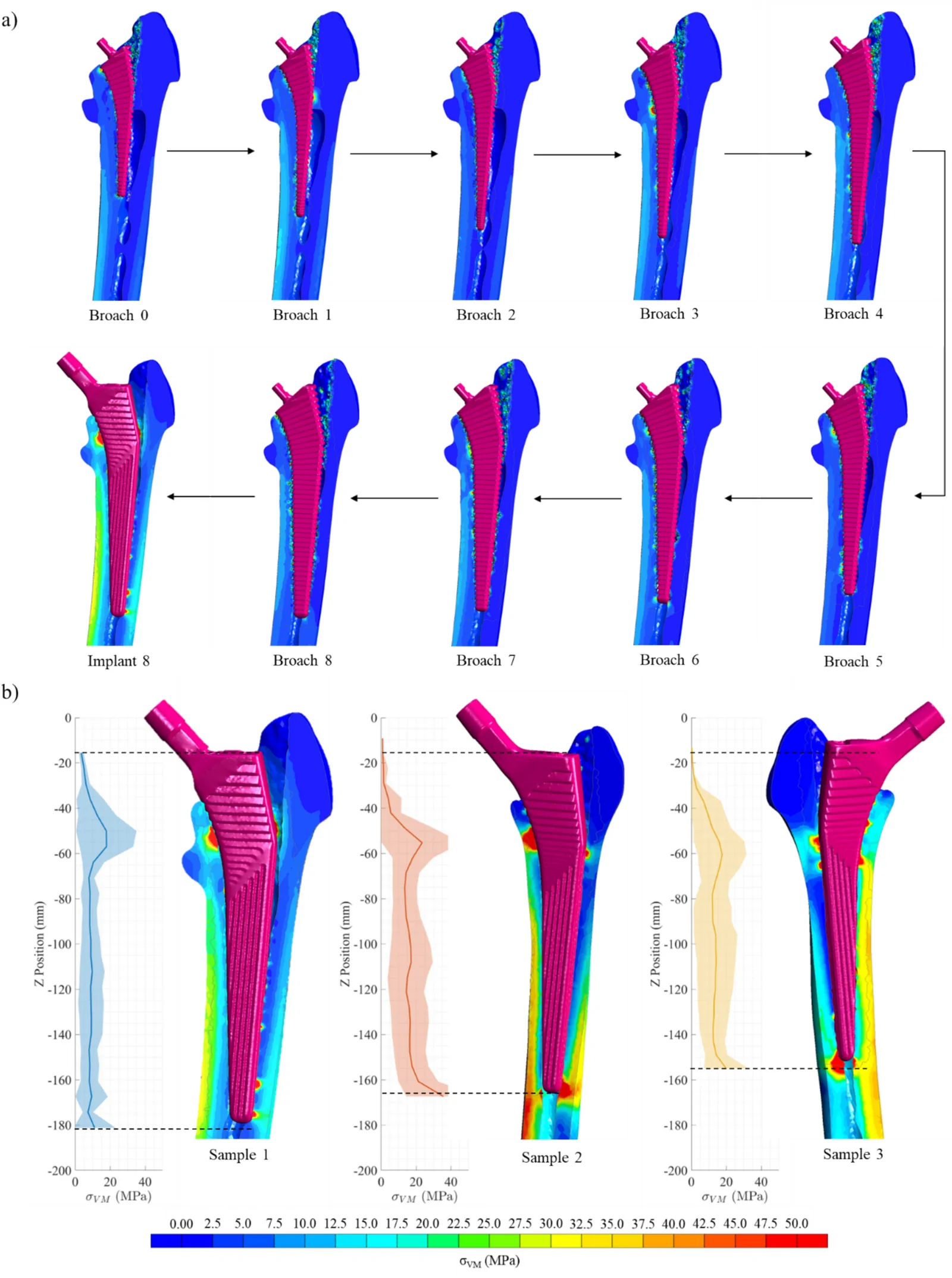
von Mises stress (*σ*_*VM*_) induced in the femurs **a** von Mises stress (*σ*_*VM*_) contours in femur sample 1 at the end of each broaching stage and after final implantation, shown from the posterior view with the implant in the nominal (planned) location, **b** von Mises stress (*σ*_*VM*_) distribution with the implant in the nominal (planned) location at the bone-implant interface (mean ± standard deviation), calculated within a 5 mm distance from the implant surface along the implant axis direction for all three femur samples. The solid lines represent the mean values, and the shaded regions indicate the standard deviation

**Fig. 4:**
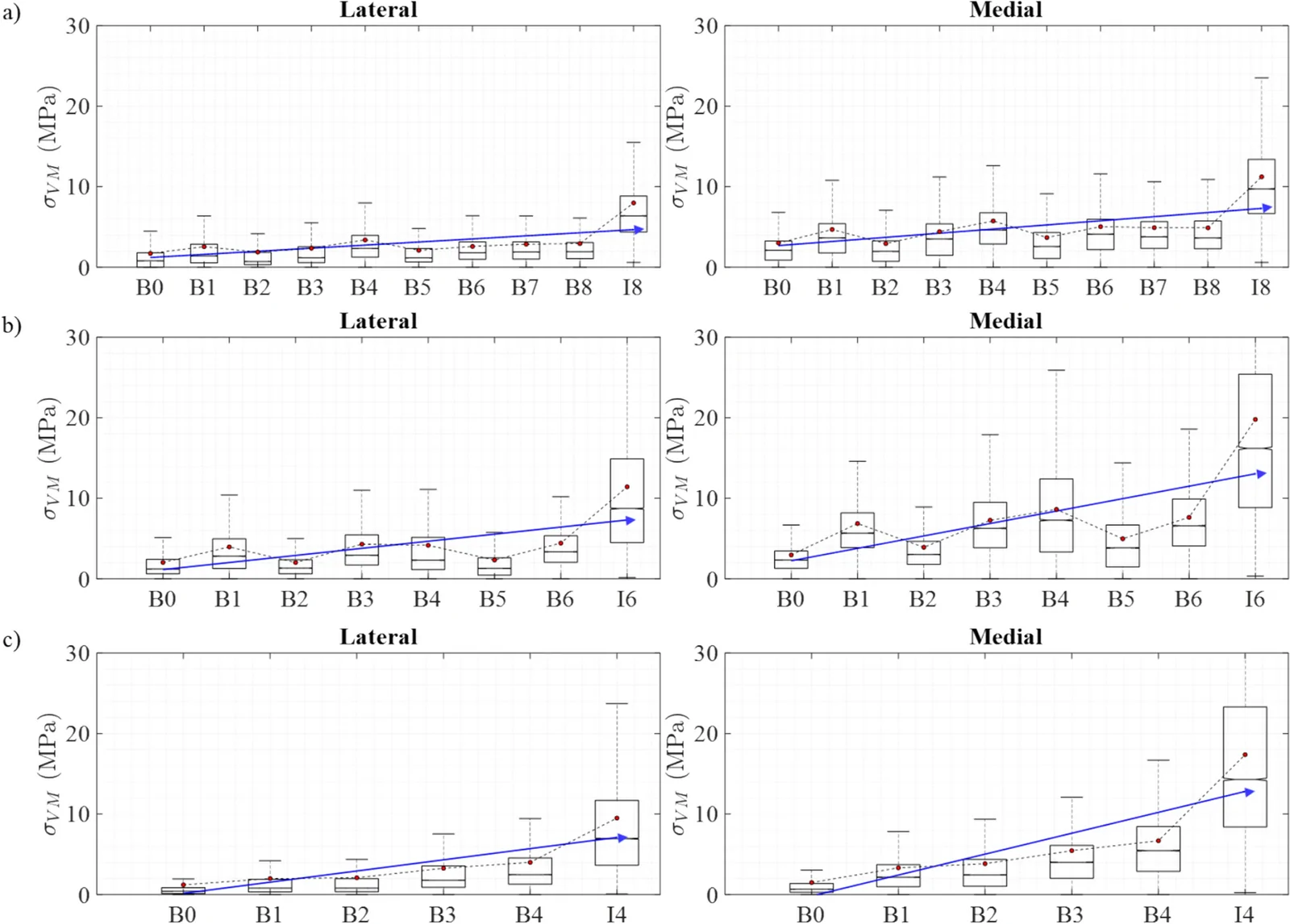
von Mises stress (*σ*_*VM*_) distribution at the bone-implant interface, calculated within a 5 mm region from the implant surface after each broaching (B) and implantation (I) stage for all three femur samples. The dotted black line connects the mean von Mises stress values at the bone-implant interface, with a blue arrow indicating the regression line and its corresponding slope: **a** femur sample 1, **b** femur sample 2, and **c** femur sample 3

Fig. 3a shows the von Mises stress (*σ*_*VM*_) distribution in femur sample 1 during broaching of each size and after final implantation, results for samples 2 and 3 are in Fig. 12 in Appendix C. Furthermore, the equivalent microstrain contours during broaching at each size and after final implantation for the three femur samples are presented in Appendix D. Fig. 3b shows that von Mises stress (*σ*_*VM*_) is generally consistent along the proximal-to-distal direction across all broach sizes and implants for all samples. However, localised stress concentrations deviate from this overall trend, particularly at the distal tip of the stem where bone-implant contact is present. For example, the proximal stress peak observed in femur 1 after implantation corresponds to a localised region of bone-implant contact (Fig. 3). Average von Mises stress (*σ*_*VM*_) at the bone-implant interface increased with broach size (Figs. 3b and 4), though local variations reflected bone heterogeneity. Stress distribution patterns were consistent across samples (Fig. 3b).

On the lateral side, average stress increases per broach size were 0.38 MPa (*R*^2^ = 0.413) for sample 1, 0.88 MPa (*R*^2^ = 0.434) for sample 2, and 1.39 MPa (*R*^2^ = 0.741) for sample 3. Medially, the increases were 0.51 MPa (*R*^2^ = 0.441), 1.54 MPa (*R*^2^ = 0.493), and 2.59 MPa (*R*^2^ = 0.734) for samples 1–3, respectively (Fig. 4). Medial stresses consistently exceeded lateral values (Figs. 3b and 4). Post-implantation average von Mises stress (*σ*_*VM*_) were 9.87 ± 8.92 MPa (sample 1, size 8), 14.77 ± 14.59 MPa (sample 2, size 6), and 12.40 ± 12.27 MPa (sample 3, size 4) (Fig. 3b).

### Micromotion between bone and implant post-implantation

**Fig. 5:**
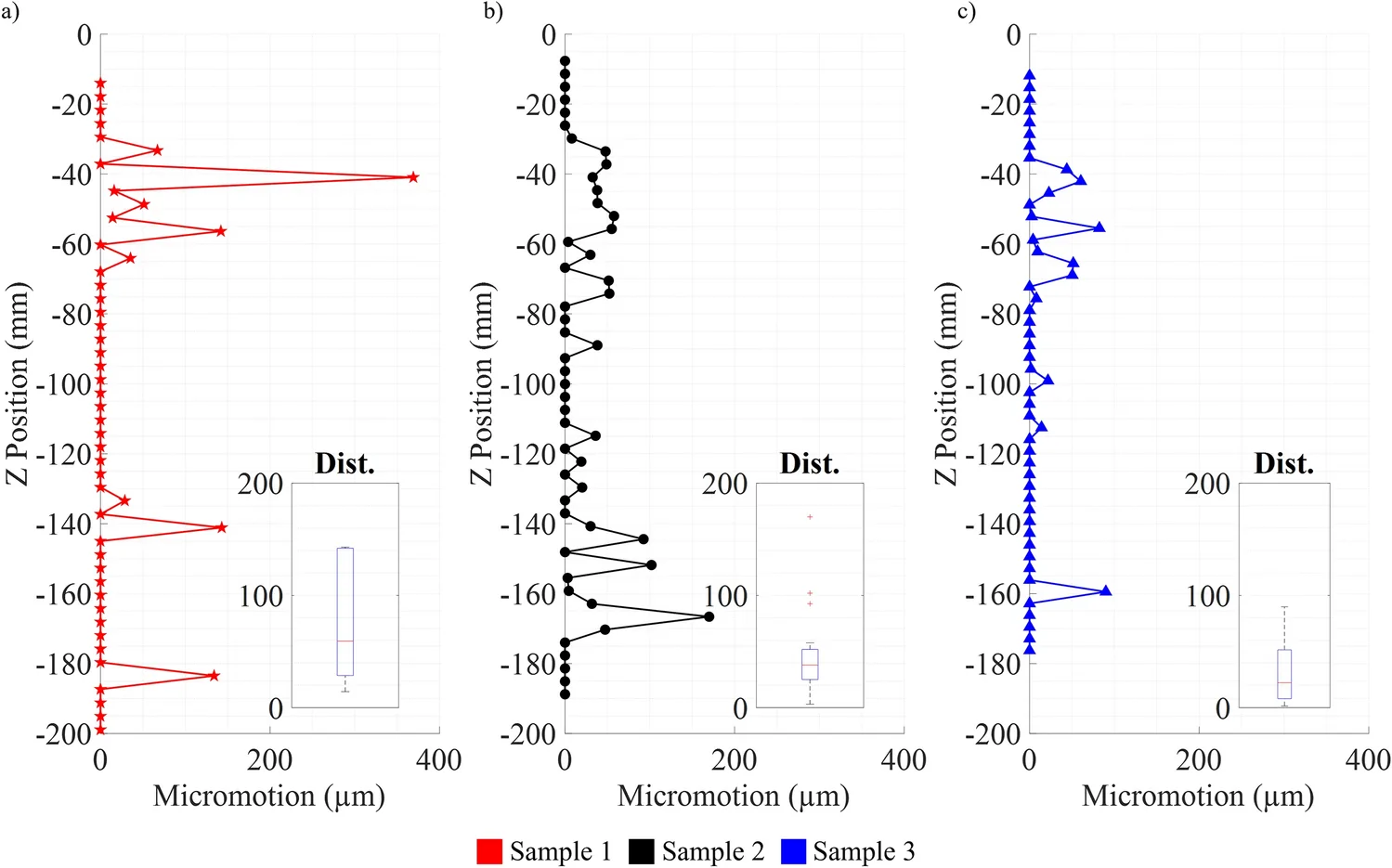
Micromotion between the bone and the implant after implantation under maximum load during daily activities along the implant axis direction (calculated within a 5 mm distance from the implant surface along the implant axis), along with the micromotion distribution, is shown for: **a** femur sample 1, **b** femur sample 2, and **c** femur sample 3, represented in different colours

Micromotion along the implant axis was heterogeneous, with regions of higher bone stress generally correlating with areas of increased micromotion across the three femur samples, particularly in Gruen zones 1 and 7 [24, 42, 55] in the proximal region of the femur (Fig. 5). This was most pronounced in femur sample 1, where elevated proximal von Mises stress (*σ*_*VM*_) (red regions in Fig. 3b) coincided with increased micromotion between − 40 and − 60 mm along the implant axis (Fig. 5a). Similar trends were observed in samples 2 and 3. The average micromotion across the three femur samples was 78.04 ± 98.05 µm (0–369 µm) for sample 1 (size 8), 40.92 ± 38.16 µm (0–170 µm) for sample 2 (size 6), and 29.10 ± 28.45 µm (0–89.9 µm) for sample 3 (size 4).

### Study 2 results: Validation of the proposed FEA

**Fig. 6:**
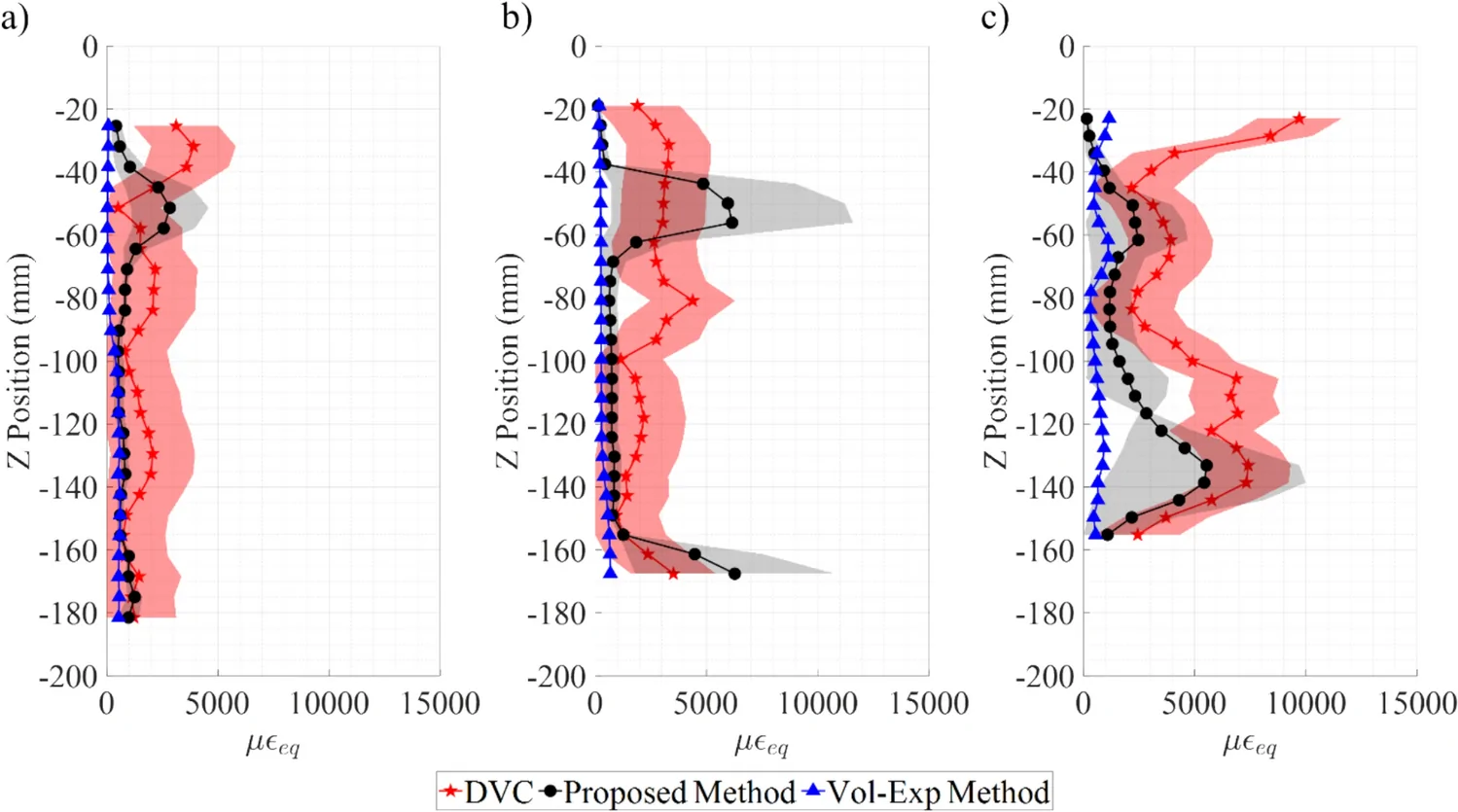
Comparison of total equivalent microstrain (*µε*_*eq*_) along the implant axis at the bone-implant interface (calculated within a 5 mm distance from the implant surface along the implant axis direction), from proximal to distal ends, measured using DVC [38] and predicted by *Proposed FEA Method* and *Volumetric Expansion Method* post-implantation: **a** femur sample 1, **b** femur sample 2, and **c** femur sample 3. Red lines indicate DVC mean values with shaded 95% confidence intervals for measurement uncertainty, black lines show Proposed FEA Method mean predictions with shaded 95% confidence intervals representing  implant positioning uncertainty, and blue lines indicate Volumetric Expansion Method predictions

*Proposed FEA Method* generally predicted slightly lower strain values than those measured by DVC, with notable deviations at specific locations along the implant axis from proximal to distal ends (Fig. 6). The mean differences in equivalent microstrain (*μɛ*_*eq*_) between *Proposed FEA Method* and DVC at each location were 1016.15 ± 878.57 for sample 1, 1755.48 ± 1028.36 for sample 2, and 2725.82 ± 2145.15 for sample 3. Although direct pointwise comparisons between DVC and the proposed FEA method are limited, the equivalent microstrain (*μɛ*_*eq*_) predicted by the proposed FEA method (black shaded region in Fig. 6) overlaps with the DVC measurements within their reported measurement uncertainty (red shaded region in Fig. 6) [42] in many regions along the bone-implant interface and along the implant axis, although some regions do not show any overlap. This overlap is particularly pronounced near the bone-implant interface along the implant axis, especially for femur sample 3, and is comparable across the other samples. Potential explanations for the observed discrepancies are discussed in subsequent sections. The uncertainties in strain from both the proposed FEA method, reflecting implant positioning variation, and the DVC measurements are shown as mean values (solid lines) with their 95% confidence intervals represented as shaded regions.

### Study 3 results: Comparison with the literature

**Fig. 7:**
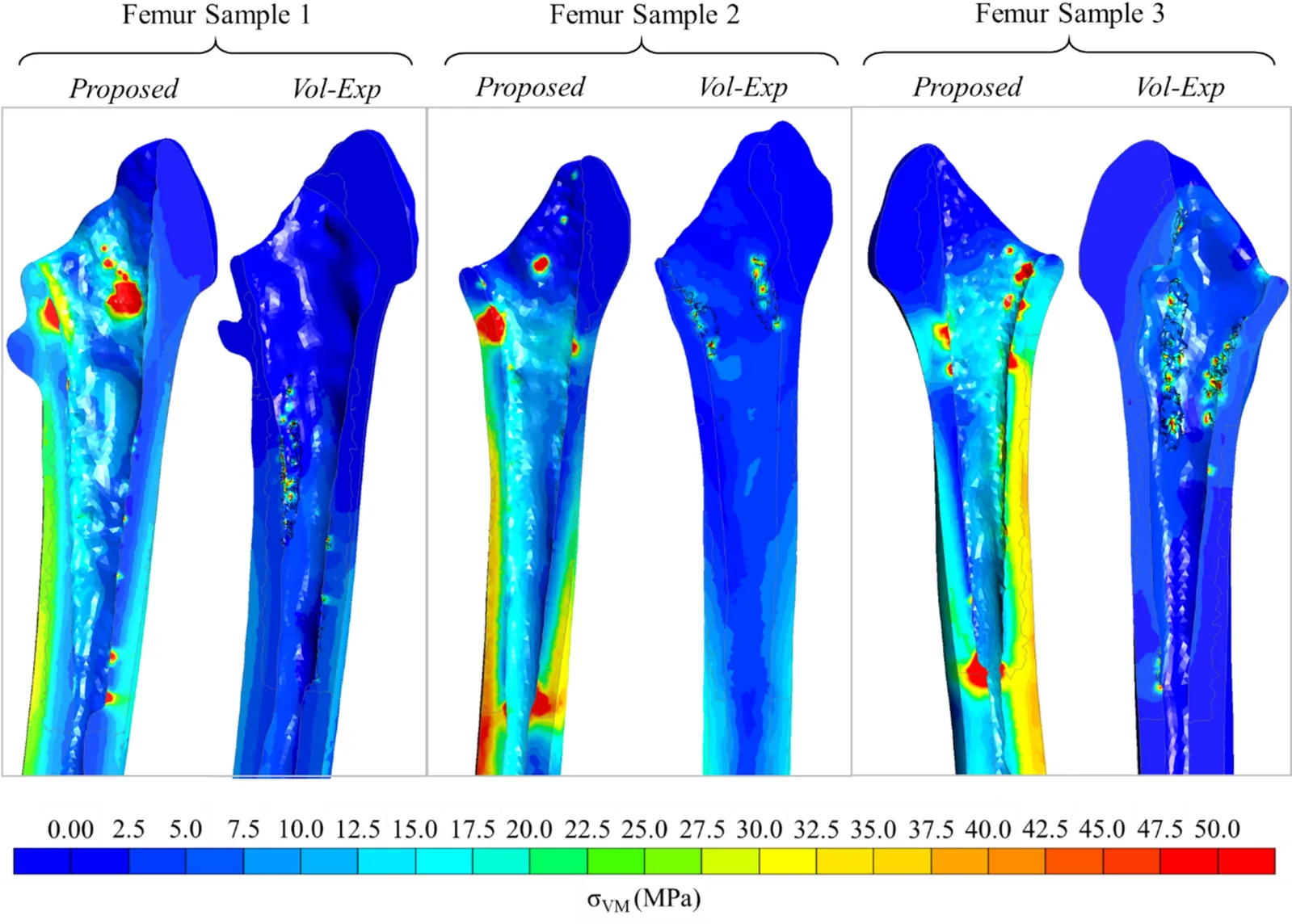
von Mises stress (*σ*_*VM*_) contours in the first femur at the end of implantation (posterior view), comparing results from two FEA models: FEA framework proposed in this study (*Proposed FEA Method*), and *Volumetric Expansion Method* approach. Results are shown for all three femur samples from the case study

The stress distribution predicted by *Volumetric Expansion Method* differed from that of *Proposed FEA Method*, generally exhibiting lower stress values than the proposed framework (Fig. 7). Stress contours obtained using the *Volumetric Expansion Method* showed less variation compared with those from the *Proposed FEA Method* (Fig. 7). Additionally, the spatial strain patterns predicted by *Volumetric Expansion Method* did not align with the trends measured by DVC (Fig. 6). The mean differences between *Volumetric Expansion Method* and DVC-evaluated equivalent microstrain (*με*_*eq*_) at each location were 2077.68 ± 1010.75 for sample 1, 2841.38 ± 921.74 for sample 2, and 4904.25 ± 2014.65 for sample 3, approximately twice those observed for the proposed model (Fig. 6).

### Study 4 results: Bone material property sensitivity on FEA

**Fig. 8:**
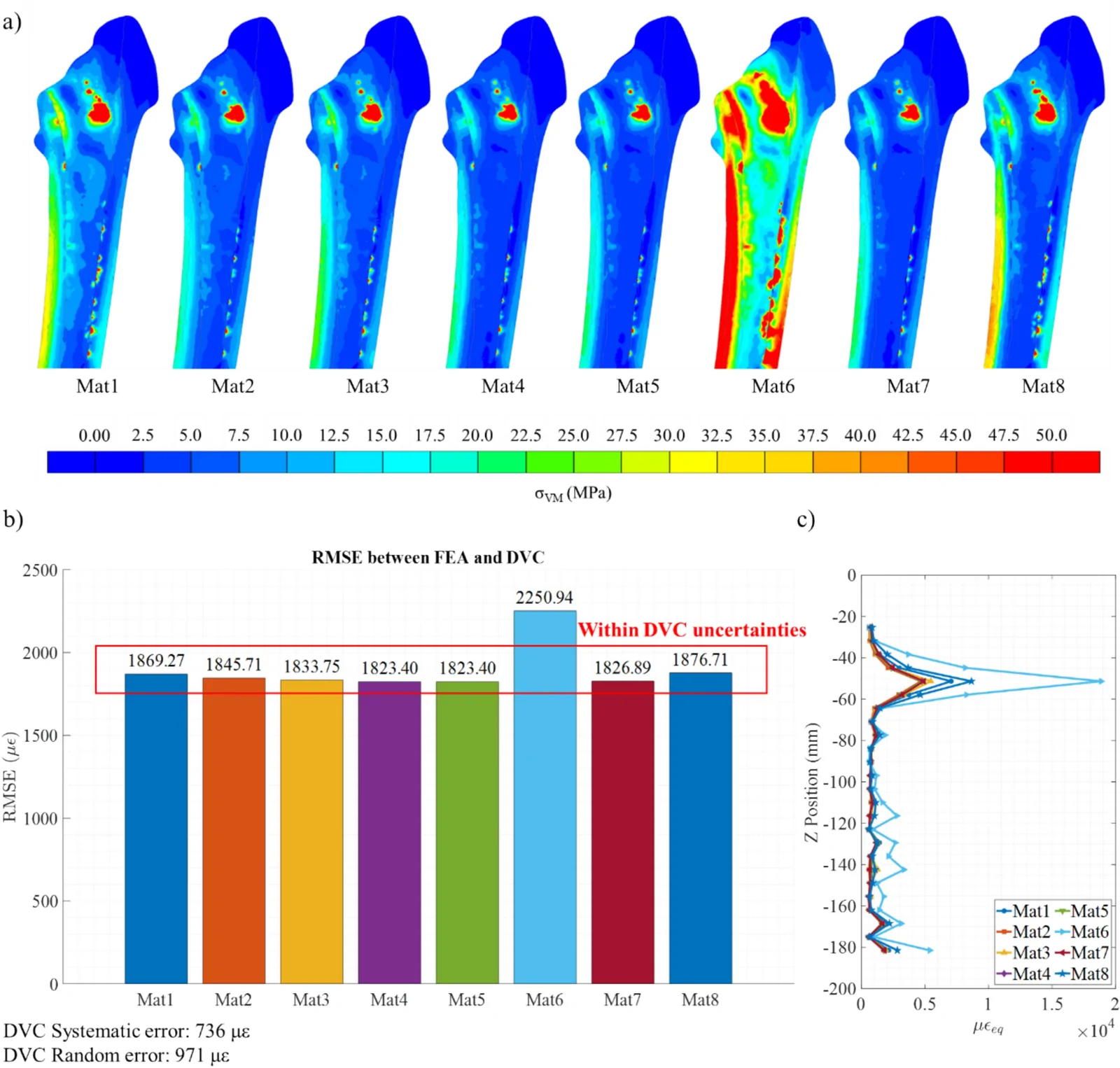
Bone material property sensitivity on FEA results: **a** von Mises stress (*σ*_*VM*_) contours in the first femur at the end of implantation (posterior view), comparing different elastic modulus-density (*E-ρ*) relationships; **b** RMSE between FEA-evaluated total equivalent strain (*με*_*eq*_) and DVC-measured strain; **c** total equivalent strain (*με*_*eq*_) at the bone-implant interface calculated within a 5 mm distance from the implant surface along the implant axis direction

**Fig. 9:**
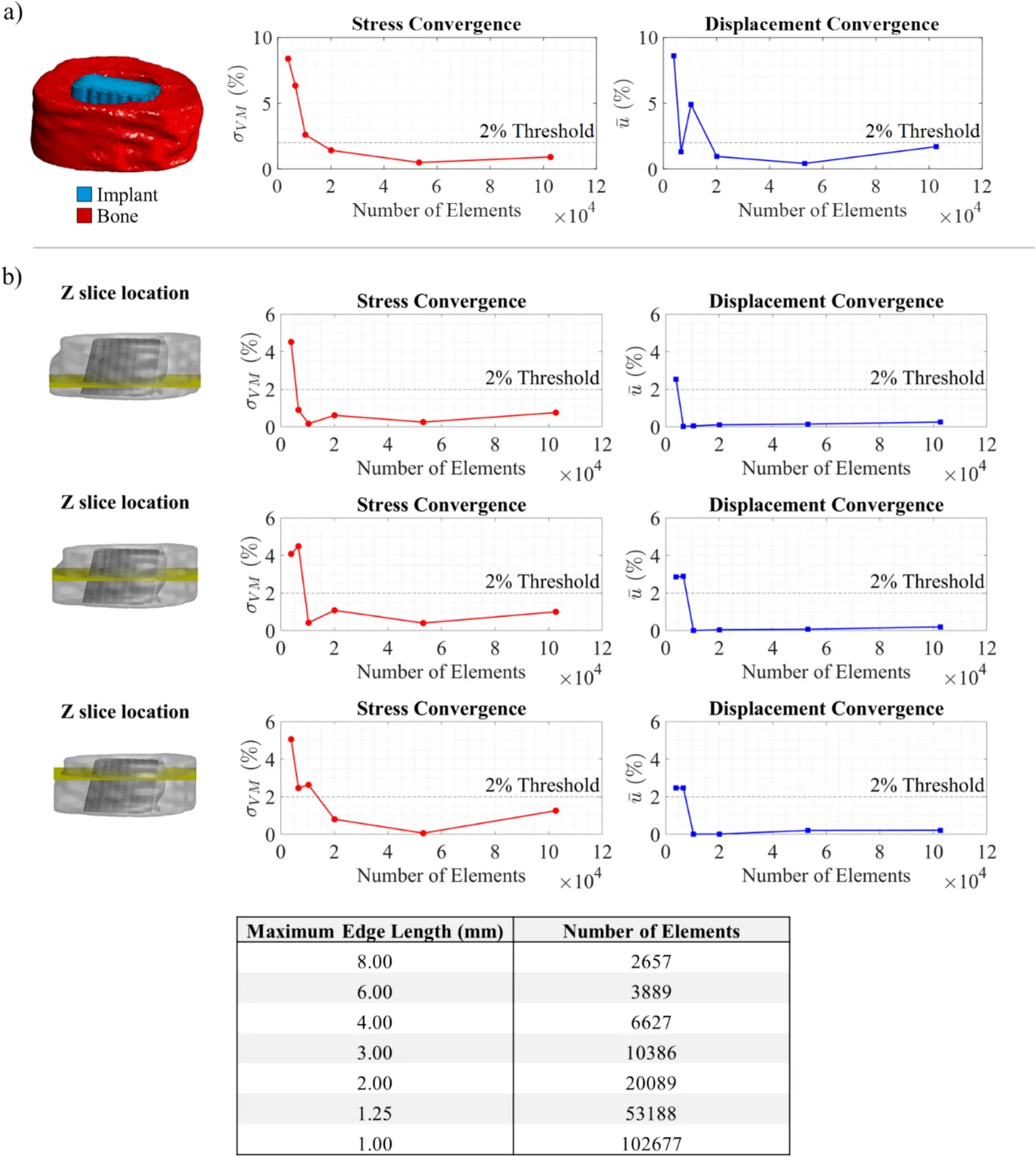
FEA mesh convergence results: **a** convergence of average von Mises stress and average displacement magnitude, and **b** convergence within 5 mm of the implant surface along the implant axis, with the evaluated locations marked by yellow planes

**Fig. 10:**
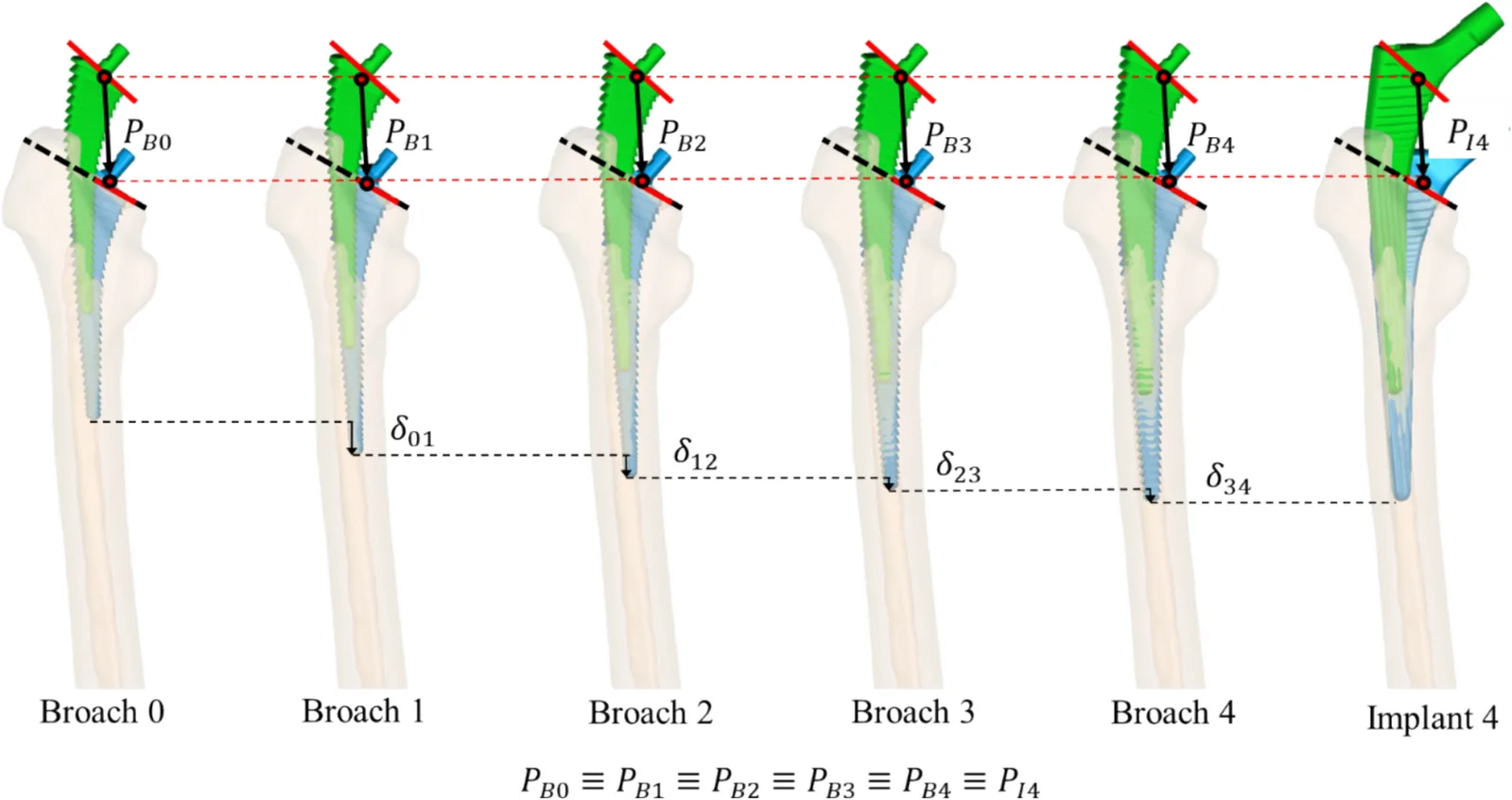
Defined insertion paths for the broaches and implant in femur sample 3. The initial and final positions of all broach and implant neck plane centres are marked in red dots with black borders, along with the corresponding paths for broaches ${P}_{B0},{P}_{B1},\dots ,{P}_{B4}$ and the implant ${P}_{I4}$. Differences in insertion depth between consecutive broach sizes are indicated by black arrows and labelled ${\delta }_{01},{\delta }_{12},\dots ,{\delta }_{34}$. The broach and implant neck planes are highlighted in red, and the femoral neck resection plane is shown as a black dotted line

**Fig. 11:**
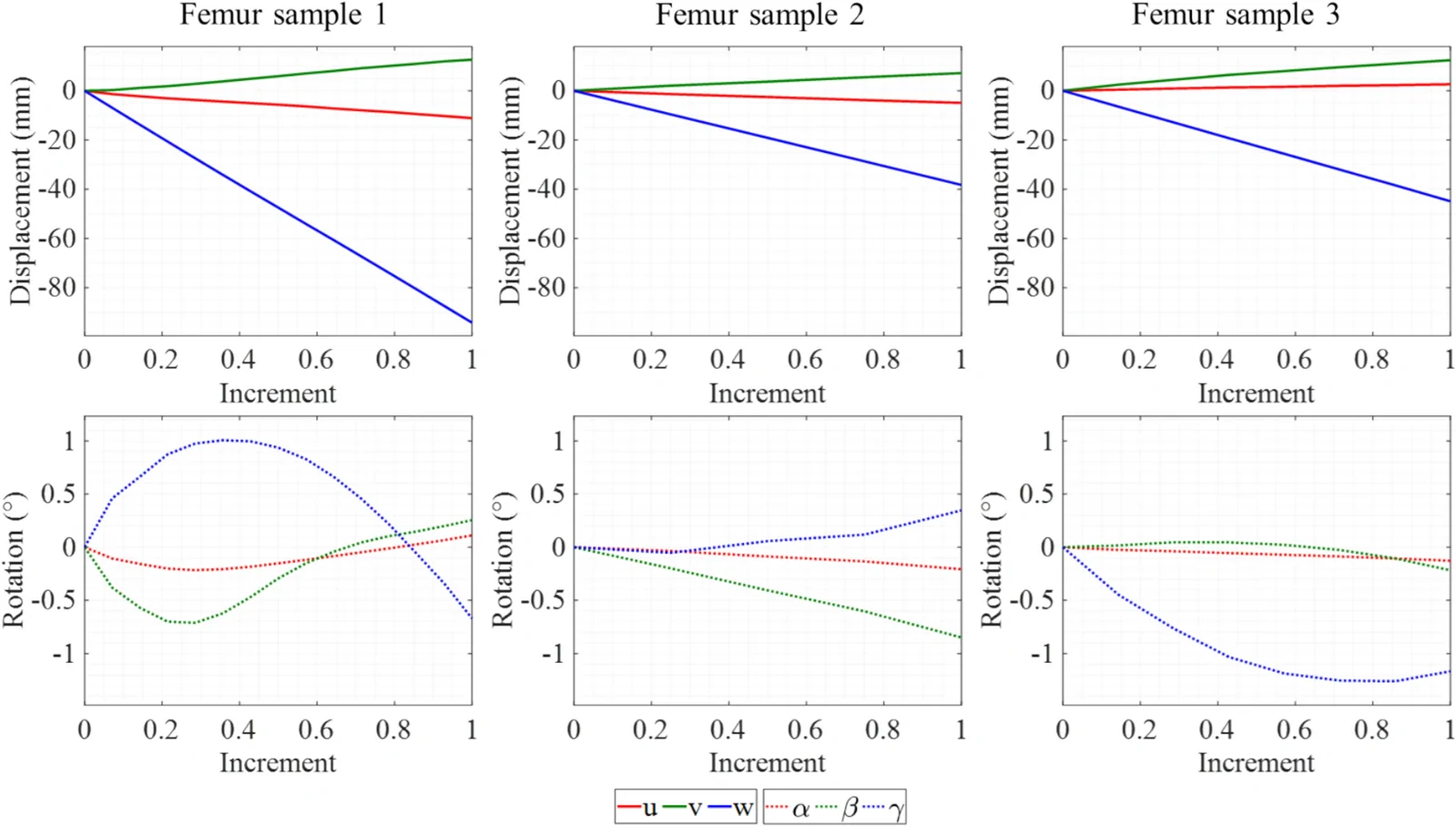
Prescribed displacement components (u, v, w) and Euler angles rotation components (γ, β, α) of the broach/implant along its defined path into the femur relative to the initial position of the broach/implant

**Fig. 12:**
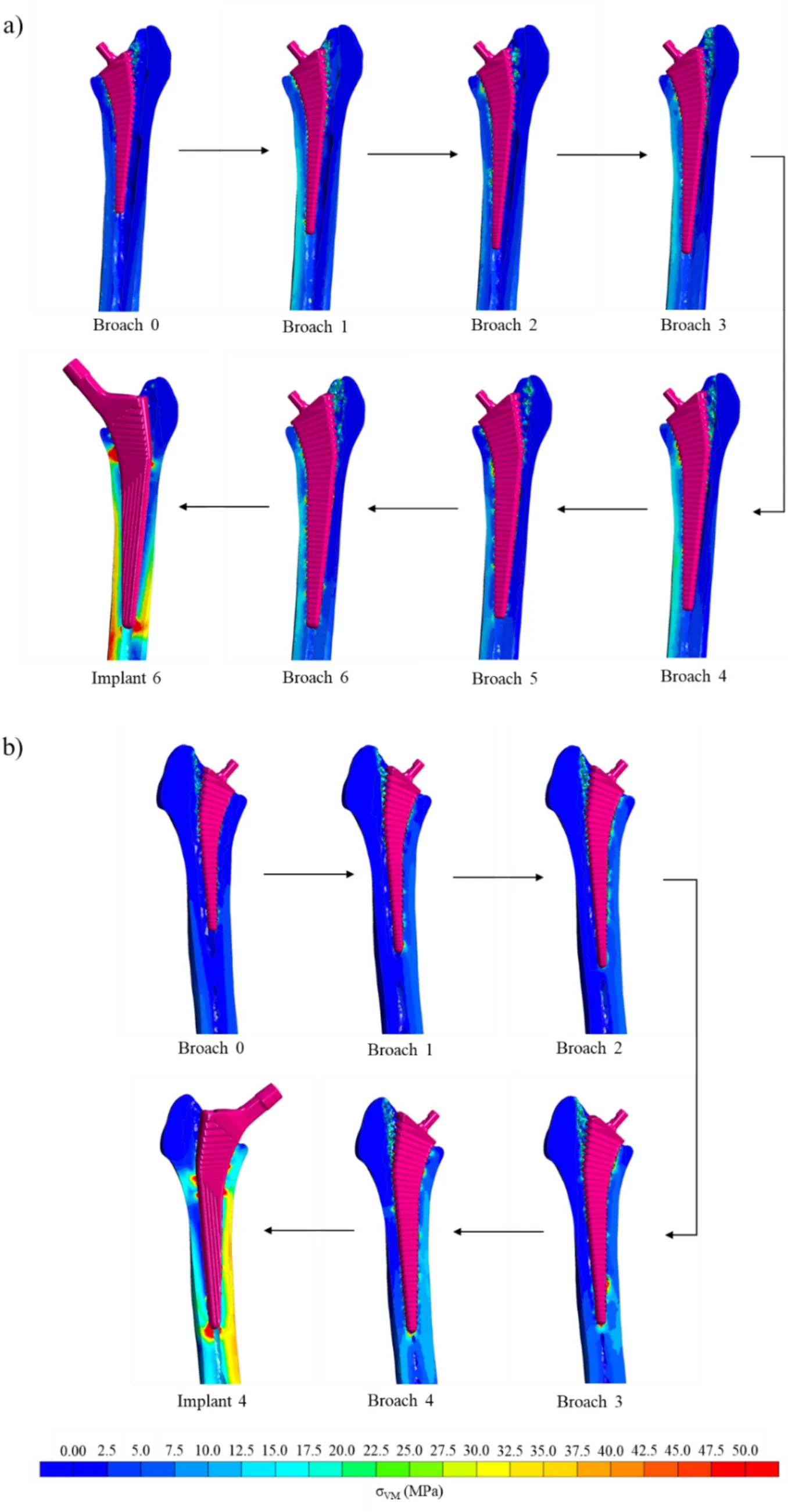
von Mises stress (σ_VM_) contours in the femur samples at the end of broaching and implantation (posterior view) with the implant in the nominal (planned) location: **a** femur sample 2, **b** femur sample 3

Stress contours from eight material models were generally consistent, except Mat6, which showed markedly higher stress (Fig. 8a). RMSE between FEA-predicted equivalent microstrain (*με*_*eq*_) and DVC-measured strain ranged from 1823.40 to 2250.94 µε (Fig. 8b). All models except Mat6 had RMSE differences within DVC uncertainty bounds [42], whereas Mat6 exceeded these limits (Fig. 8b). The total equivalent strain (*με*_*eq*_) at the bone-implant interface, calculated within a 5 mm distance from the implant surface along the implant axis, was higher for Mat6 compared with the other material models, in contrast, the remaining models showed very similar and overlapping strain values, as observed in Fig. 8c.

## Discussion

Simulation models capturing the stepwise broaching and implantation process, critical for evaluating IOPPFs and primary stability related to aseptic loosening in uncemented THA, are largely absent from the literature. To the authors’ knowledge, this is the first FEA framework to replicate the complete insertion process, including stepwise broaching and implantation, while also enabling primary stability assessment. The main contributions are:

- **Insertion Path Definition**: Realistic broach and implant paths were defined to simulate THA insertion conditions, addressing a key gap and supporting future investigations.
- **FEA Framework Development**: The framework replicated the actual uncemented THA procedure by simulating insertion, including stepwise broaching and implantation, and subsequent stability assessment, features that were not included in previous models.
- **Stress Response During Broaching**: Simulations using three cadaveric femoral sample showed average increase in von Mises stress (*σ*_*VM*_) of 0.39–2.59 MPa with each broach size, reflecting progressive trabecular bone compaction. Post-implantation micromotion ranged from 29.10 to 78.04 µm, remaining below the osseointegration threshold under ADL loads.
- **FEA Experimental Validation**: The model-predicted strain values after implantation were validated against experimentally measured strain data from a cadaveric study using micro-CT-based DVC analysis, with mean strain differences (*µε*_*eq*_) ranging from 1016.15 to 2725.82.
- **Comparison with Existing Models**: The *Proposed FEA Method* produced strain values that more closely matched DVC measurements from the cadaveric study compared to the *Volumetric Expansion Method* [17]. The *Volumetric Expansion Method* exhibited more uniform stress contours but less accurate distributions, resulting in nearly double the error compared to DVC measurements when compared to the proposed framework (*Proposed FEA Method*).
- **Material Model Sensitivity**: The framework exhibited limited sensitivity to eight *E-ρ* material models from the literature, all producing consistent/similar results except Mat6, which exceeded DVC uncertainty bounds.

Across three femur samples, average von Mises stress (*σ*_*VM*_) in bone ranged from 9.87 to 14.77 MPa (Fig. 3b), with equivalent microstrain (*με*_*eq*_) values consistent among samples and comparable to experimentally measured implantation surface strains reported in the literature [56]. The induced von Mises stress (*σ*_*VM*_) was generally consistent along the proximal-to-distal direction across all broach sizes and implants for all samples. Localised stress concentrations deviated from this overall trend, corresponding to regions of bone-implant contact, particularly at the distal tip of the stem. This suggests enhanced distal fixation, consistent with the three-point contact mechanism described by Seemala et al. [42]. Notably, a proximal stress spike occurred in femur sample 1 post-implantation, attributed to localised bone-implant contact (Fig. 3), aligning with previous DVC-based strain findings [42] (Fig. 6). These localised stress concentrations could lead to differing levels of bone remodeling. If the difference in mechanical stimulus (strain energy density) between the intact femur and the post-implantation state exceeds the lazy zone due to stress concentrations, it may trigger bone remodeling [57, 58]. Specifically, when the metallic implant bears a greater load than the surrounding bone and the resulting mechanical stimulus surpasses the lazy zone, bone resorption may occur, increasing the risk of post-THA PPF and aseptic loosening. Therefore, the proposed FEA simulation provides a framework for comparing the extent of localised high-stress regions, stress-strain throughout the bone due to the surgical process, as well as associated micromotion and fracture risk, to support the selection of the appropriate implant size. Although this analysis is beyond the scope of the current study, it represents an important direction for future research by providing the initial conditions of bone-implant interface stresses following the surgical procedure.

During broaching, average von Mises stress (*σ*_*VM*_) at the bone–implant interface increased by 0.39–2.59 MPa per broach size (Fig. 4), indicating trabecular bone removal and compaction that support press-fit fixation. Medial stress and strain were consistently higher and increased more rapidly than lateral values, reflecting greater medial trabecular compaction. No significant anterior-posterior differences were observed.

The *Proposed FEA Method* enabled assessment of primary prosthesis stability, predicting micromotions under ADL loading of 78.04 ± 98.05 µm (Sample 1), 40.92 ± 38.16 µm (Sample 2), and 29.10 ± 28.45 µm (Sample 3) (Fig. 5). Most values were below the 150 µm threshold favourable for osseointegration [48–50]. Although the literature reports an average micromotion of 112 ± 176 µm for osseointegrated implants, with overlap between successful and failed cases [47], all implantations in this study were performed by a surgeon with over 25 years of experience, who judged the procedures successful, and the implants stable based on their subjective assessment. The lower predicted micromotions compared to literature [47] further support primary stability. The assessment of micromotion using the proposed FEA method relative to established thresholds should be interpreted as a verification rather than a validation. These results demonstrate the framework’s effectiveness in assessing *in-silico* implant stability following uncemented THA and its potential to predict aseptic loosening, thereby supporting surgeons in preoperative planning and implant selection, which is currently lacking in the literature Furthermore, regions of higher bone stress, particularly in Gruen zones 1 and 7 [24, 42, 55] in the proximal region of the femur, were generally associated with increased micromotion (Figs. 3 and 5). However, direct causation cannot be established due to the limited sample size in this study. Further research is needed to determine whether minimising local stress, particularly in the proximal femur, where greater osseointegration is desired, is critical for implant design and surgical technique, and whether reducing stress in these regions could enhance initial stability and promote long-term biological fixation.

The *proposed FEA Method* was validated by comparing FEA-predicted strains with those measured using DVC. Direct pointwise comparisons between the two techniques are inherently limited due to differences in imaging modalities: the FEA model was constructed using medical-CT data (voxel size: 0.5 × 0.5 × 0.625 mm), whereas the DVC measurements were obtained from high-resolution μCT images (voxel size: 70 × 70 × 70 μm). Consequently, μCT could resolve the finer trabecular microstructure and capture deformation of individual trabeculae caused by the surgical process during the experiment. In contrast, the medical-CT-based FEA model could not reproduce the same trabecular geometries due to its lower resolution. As a result, although the same cadaveric femur was used, local geometric differences in trabecular structures arose between the FEA and DVC analyses, limiting direct comparison.

Although micro-FE using μCT images by converting each scanned voxel into an FEA element [59–62] or geometry-based micro-FE on a small ROI [21] is possible, incorporating μCT data into the *proposed FEA method* is not feasible because the extremely large μCT dataset required for a full femur would result in an unmanageably high element count and impractical computational time. In addition, μCT cannot be used in vivo, making micro-FE unsuitable for patient-specific modeling, which is central to the *proposed FEA framework*. Furthermore, DVC analysis using medical-CT is not feasible because the accuracy and precision needed to capture the displacements and strains produced during the surgical process cannot be achieved with its lower resolution. Medical-CT cannot resolve the fine trabecular deformations required, and the subset size cannot be increased sufficiently to balance spatial resolution with accurate strain measurement [63].

These factors contribute to differences between the DVC-derived strain values and those computed by the proposed FEA method. The RMSE percentage differences between the DVC strains and the proposed FEA Method were 78.36%, 82.62%, and 71.00% for femur samples 1, 2, and 3, respectively. These values are comparable to prior studies comparing DVC measurements with voxel-based micro-FE under simplified compressive loading, where RMSE values ranged from 5% to 81% across mouse tibiae [59], cadaveric arm [60], vertebrae [61], and humeral head [62].

The observed discrepancies may also arise from residual errors during registration of medical-CT and μCT images, which can be affected by variations in specimen geometry and local morphological detail. Because trabecular bone is thin, slender, and naturally curved, even small mismatches in experimental and computational conditions can substantially influence local strain predictions in DVC analyses [59]. Additionally, the viscoelastic behaviour of bone, which can cause stress relaxation after broaching, may further contribute to differences between the DVC measurements and the FEA results, as this behaviour was not modelled in the FEA framework.

Despite these differences, the range of equivalent microstrain (*μɛ*_*eq*_) predicted by *Proposed FEA Method* (black shaded region in Fig. 6) overlapped with that measured by DVC within its reported measurement uncertainty (red shaded region in Fig. 6) [42] in many regions along the bone–implant interface and along the implant axis. This overlap was particularly pronounced near the bone–implant interface along the implant axis, especially for femur sample 3, and was consistent across other samples. These findings support previous observations that, despite resolution-related limitations, medical-CT-based FEA and μCT-based DVC produce comparable mean strain magnitudes (DVC (*μɛ*_*eq*_): 2449.86 ± 859.82; FEA (μɛ_*eq*_): 1702.55 ± 2015.46) and similar spatial distributions [21].

Unlike existing FEA models for uncemented THA that simulate press-fit via volumetric expansion [17] or opposing point forces [18, 19], the proposed framework explicitly modelled individual broaching insertion steps and tracks broach and implant insertion through the femur. *Volumetric Expansion Method* yielded lower-magnitude stress fields due to symmetric assumptions, oversimplifying contact mechanics and omitting anterior–posterior asymmetry seen during surgery [42]. The proposed framework better captured intraoperative mechanics, producing bone–implant interface strain patterns more consistent with DVC measurements (Fig. 6). The difference in *με*_*eq*_ between *Proposed FEA Method* and DVC was nearly half that of *Volumetric Expansion Method*’s model, reflecting stronger experimental agreement, including the observed proximal-to-distal strain gradient (Fig. 7). von Mises stress (*σ*_*VM*_) contours also differed notably between models.

FEA results showed limited sensitivity to *E-ρ* relationships; stress contours and RMSE differences between FEA and DVC strains were consistent across models and generally within DVC uncertainty bounds, except Mat6, which exhibited elevated stress, strain and RMSE beyond these limits. This indicates all evaluated *E-ρ* models, except Mat6, yield reliable, consistent outcomes, permitting flexible material property selection without significantly impacting predicted stress, strain, or study conclusions (Fig. 8).

This study has a few limitations. First, the press-fit procedure was modelled quasi-statically rather than dynamically; however, the time interval was sufficiently long to minimise inertial effects, making dynamic analysis unnecessary. Second, the uncertainty in implant positioning within the FEA framework was modelled only along the proximodistal direction, while variations in the other two directions were not considered. Incorporating these additional positional uncertainties would further enhance the predictive accuracy of the proposed framework. Third, soft tissue effects and intra-cavity pressure within the femoral canal were not included, though such pressure is minimal in an open canal. Fourth, the anisotropic and viscoelastic properties of bone and the deformable implant were not modelled, however, this is not expected to significantly affect the results or alter the conclusions drawn. Fifth, the micromotion between the bone and implant was not validated against experimentally measured micromotion. However, verification was performed based on the judgment of an orthopaedic surgeon with over 25 years of experience. Addressing these limitations in future studies could improve the generalisability of uncemented THA modeling.

In summary, this study presents a novel FEA framework that, to the authors’ knowledge, is the first to simulate the stepwise broaching and implantation process during uncemented THA using patient-specific geometry. It establishes a foundation for preoperative planning tools to evaluate IOPPF and aseptic loosening risk and to support tailored femoral implant selection for improved surgical outcomes. The framework enables the assessment of implant stability under clinically relevant loads by modeling bone–broach and bone–implant interactions. Across three femoral samples, the average post-implantation von Mises stress (*σ*_*VM*_) ranged from 9.87 to 14.77 MPa, with strain values and patterns at the bone-implant interface overlapping with experimentally measured DVC-evaluated strains from prior work. Each incremental increase in broach size resulted in a 0.39 to 2.59 MPa rise in bone stress, with higher absolute values and steeper increases observed on the medial side. Predicted micromotions (29.10–78.04 µm) remained below the 150 µm threshold typically associated with successful osseointegration, aligning with experimental outcomes confirmed by the operating surgeon. The FEA results showed limited sensitivity to the choice of *E–ρ* models, with consistent stress contours and minor RMSE differences across material models. Compared to the volume-expansion-based *Volumetric Expansion Method*, which oversimplifies surgical mechanics, the developed framework more closely replicated DVC-observed strain distributions, reducing prediction errors by nearly half. Overall, the FEA framework developed in this study establishes a foundation for future computational tools aimed at supporting personalised pre-surgical planning. These tools could assist in selecting the optimal stem type and size for a patient based on both bone anatomy and bone quality/density. Such tools could enable risk estimation of IOPPFs during femoral broaching and implantation, as well as assessment of micromotion to evaluate the risk of aseptic loosening, areas that remain largely underexplored in current literature and clinical practice.

## Electronic supplementary material

Below is the link to the electronic supplementary material.

Supplementary file1 (MP4 29061 KB)

## Figures and Tables

**Fig. 13:**
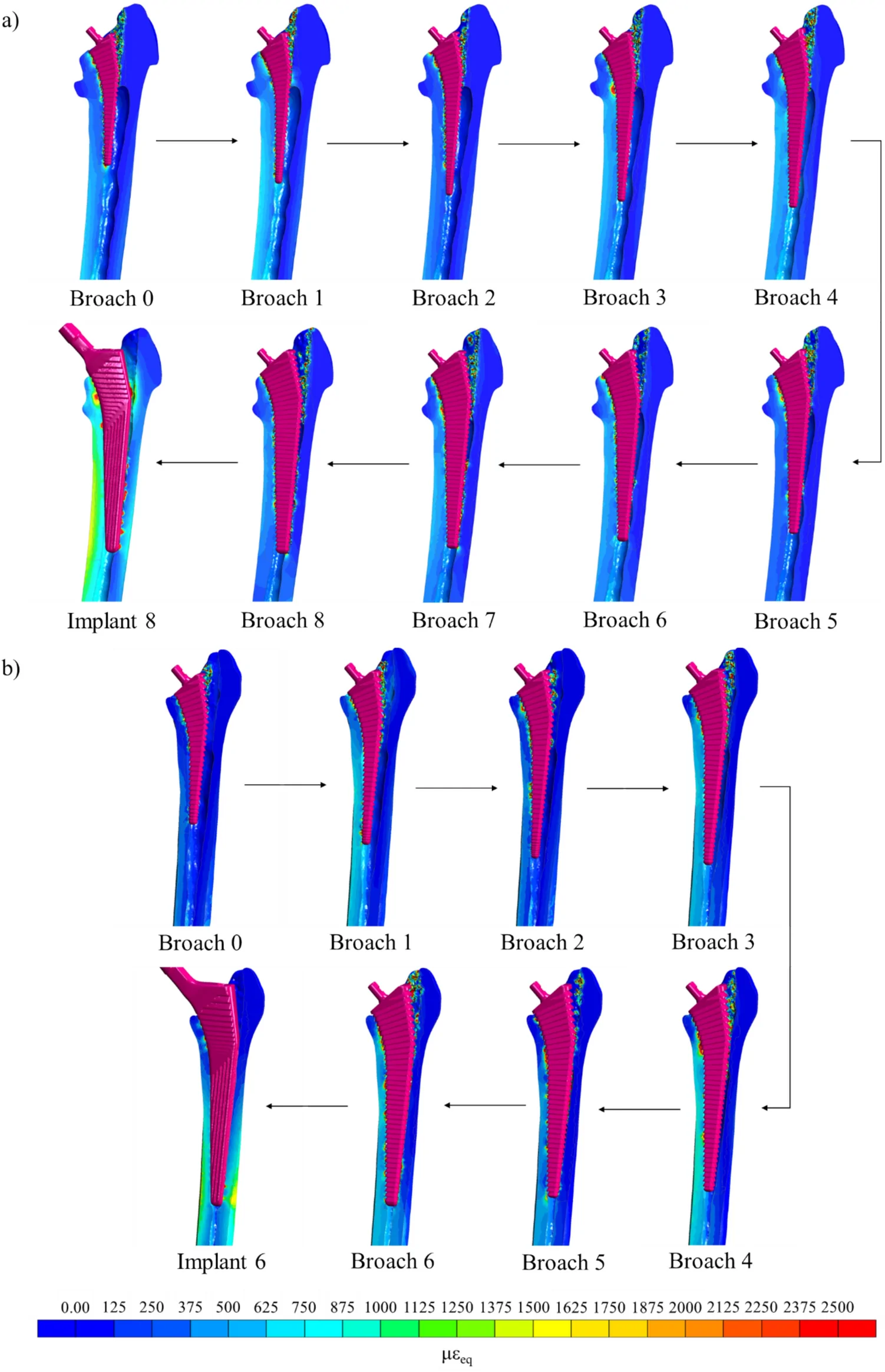

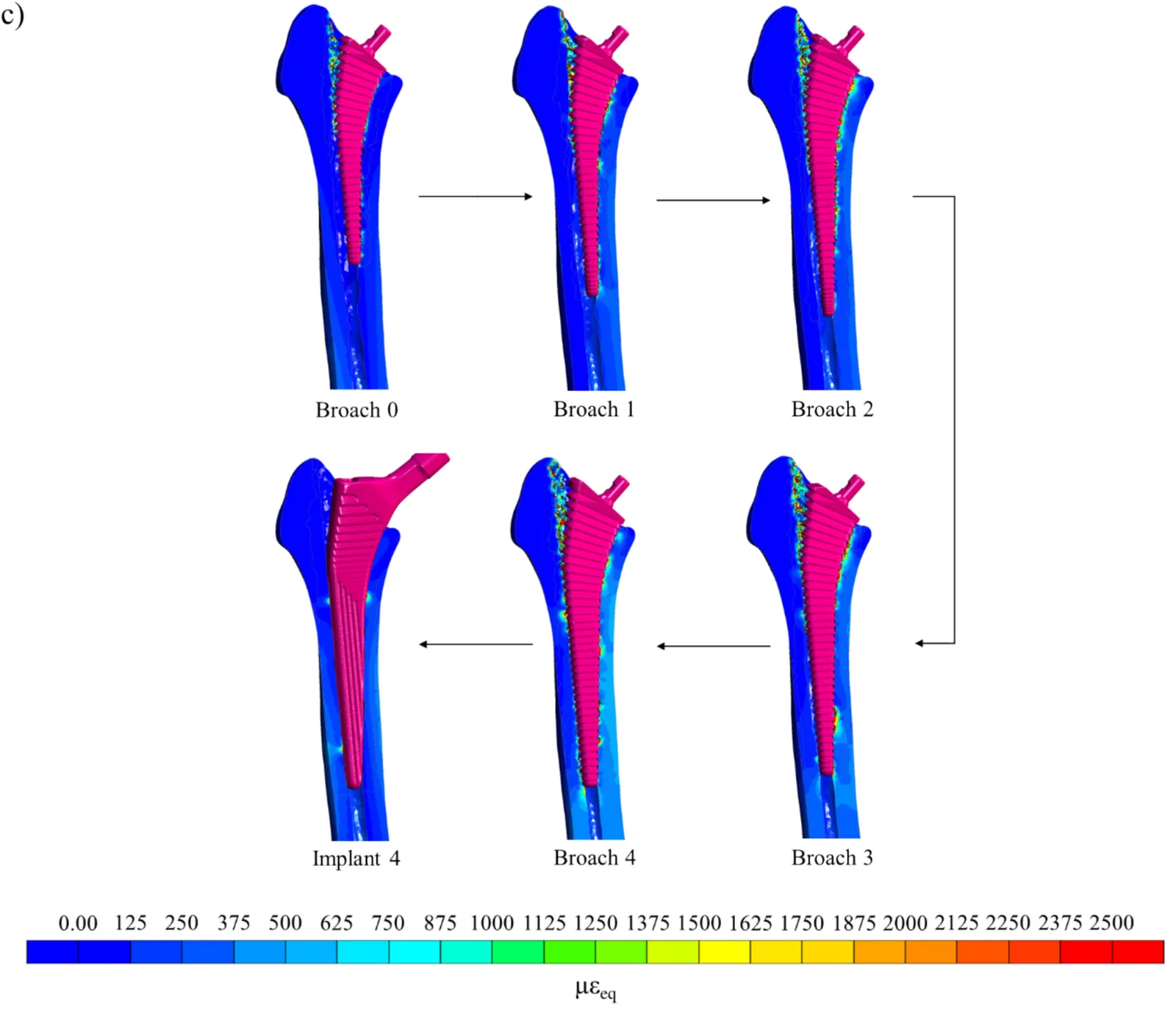
Equivalent microstrain (*µε*_*eq*_) contours in the femur samples at the end of broaching and implantation (posterior view) with the implant in the nominal (planned) location: **a** femur sample 1, **b** femur sample 2, **c** femur sample 3

## Mesh convergence study

A region of interest (ROI) near the lesser trochanter, characterised by a high bone–implant contact area [42], was selected for detailed analysis. Displacement and stress convergence around the bone-implant interface were evaluated, following the procedures described in “Broaches, implants and patient-specific femur geometry”–”Material property definition” Sects., at multiple locations along the implant axis. Eight FEA models with maximum element edge lengths of 8, 6, 4, 3, 2, 1.25, and 1 mm were analysed to determine the optimal mesh size for the case studies.

The relative percentage change between two consecutive mesh sizes was calculated with respect to the finest mesh, and a convergence threshold of 2% was applied for both stress and displacement. Convergence was achieved with approximately 20,000 elements, corresponding to a maximum edge length of less than 2 mm. The converged stress and displacement values are indicated by the dotted horizontal lines. Based on these findings, a 10-node tetrahedral mesh with a maximum edge length of 2 mm was selected for all subsequent FEA (Fig. 9).

## Appendix B

Figure 10 illustrates the defined insertion paths for the broaches and the implant in femur sample 3. The initial and final positions of all broaches and the implant are the same, as shown by the broach/implant neck-plane centres (red dots with black borders) and the red dotted horizontal lines representing transverse planes marking identical initial and final positions. This holds true for any arbitrary point on the proximal broach or implant, not just the neck-plane centre, because the motion is rigid-body. Therefore, the displacement of these points between the initial and final positions is identical. Likewise, the initial and final orientations of the broach and implant neck planes are identical, as highlighted by the red planes in Fig. 10. This alignment arises because both the broaches and the implant were positioned using the same proximal features that interface with the broach handle. Since the handle attaches consistently across all broach sizes and the implant, and its initial pose is identical during surgery, the initial orientation and position are the same for all broaches and for the implant. Insertion typically stops when the neck plane of the broach or implant becomes flush with the femoral neck resection plane during surgery. As shown in Fig. 10, the femoral neck resection plane is indicated by the black dotted line, while the broach and implant neck planes are shown in red. This constraint ensures that the final neck plane location is identical across all broach sizes and the implant, thereby making the final implant position the same for all broach sizes and for the implant. Therefore, the FEA boundary conditions applied along the paths described in Sect. “Broach and implant path definition” for broaches ${P}_{B0},{P}_{B1},\dots ,{P}_{B4}$ and the implant ${P}_{I4}$ are identical across instruments for femur sample 3.

The observed variation in insertion depth (${\delta }_{01},{\delta }_{12},\dots ,{\delta }_{34}$) results solely from differences in the lengths and geometries of the broaches and implant. The complete defined insertion path within the femoral cavity for both broaches and the implant is presented in Fig. 11.

## Appendix C

See Fig. 12.

## Appendix D

See Fig. 13.

## References

[1] Tudor, F. S., J. R. Donaldson, S. R. Rodriguez-Elizalde, and H. U. Cameron. Long-term comparison of porous versus hydroxyapatite coated sleeve of a modular cementless femoral stem (SROM) in primary total hip arthroplasty. *J Arthroplasty*. 30(10):1777–1780, 2015. 10.1016/j.arth.2015.04.031 25971533

[2] Viceconti, M., G. Brusi, A. Pancanti, and L. Cristofolini. Primary stability of an anatomical cementless hip stem: a statistical analysis. *J Biomech*. 39(7):1169–1179, 2006. 10.1016/j.jbiomech.2005.03.024 15927191

[3] Maggs, J., and M. Wilson. The relative merits of cemented and uncemented prostheses in total hip arthroplasty. *Indian J Orthop*. 51(4):377–385, 2017. 10.4103/ortho.IJOrtho_405_16 PMC5525518 28790466

[4] Huddleston, H. D. Femoral lysis after cemented hip arthroplasty. *J Arthroplasty*. 3(4):285–297, 1988. 10.1016/s0883-5403(88)80027-5 3071572

[5] Chareancholvanich, K., et al. In vitro stability of cemented and cementless femoral stems with compaction. *Clin Orthop Relat Res*. 394:290–302, 2002. 10.1097/00003086-200201000-00034 11795746

[6] Achakri, H., et al., National Joint Registry annual reports, in The National Joint Registry 21st annual report 2024. 2023, National Joint Registry © National Joint Registry 2024: London. 40198011

[7] Kurtz, S. M., et al. Future young patient demand for primary and revision joint replacement: national projections from 2010 to 2030. *Clin Orthop Relat Res*. 467(10):2606–2612, 2009. 10.1007/s11999-009-0834-6 PMC2745453 19360453

[8] Lamb, J. N., et al. Patient and implant survival following intraoperative periprosthetic femoral fractures during primary total hip arthroplasty: an analysis from the national joint registry for England, Wales, Northern Ireland and the Isle of Man. *Bone Joint J*. 101-B(10):1199–1208, 2019. 10.1302/0301-620X.101B10.BJJ-2018-1596.R1 31564148

[9] Carli, A. V., J. J. Negus, and F. S. Haddad. Periprosthetic femoral fractures and trying to avoid them: what is the contribution of femoral component design to the increased risk of periprosthetic femoral fracture? *Bone Joint J*. 99-B:50–59, 2017. 10.1302/0301-620X.99B1.BJJ-2016-0220.R1 28042119

[10] Holley, K., et al. Periprosthetic fractures of the femur after hip arthroplasty: an analysis of 99 patients. *HSS J*. 3(2):190–197, 2007. 10.1007/s11420-007-9045-4 PMC2504263 18751793

[11] Hu, K., et al. Periprosthetic fractures may be more likely in cementless femoral stems with sharp edges. *Ir J Med Sci*. 179(3):417–421, 2010. 10.1007/s11845-009-0442-1 19847592

[12] Lindberg-Larsen, M., et al. Increased risk of intraoperative and early postoperative periprosthetic femoral fracture with uncemented stems. *Acta Orthopaedica*. 88(4):390–394, 2017. 10.1080/17453674.2017.1302908 PMC5499329 28290738

[13] Carli, A. V., J. J. Negus, and F. S. Haddad. Periprosthetic femoral fractures and trying to avoid them: what is the contribution of femoral component design to the increased risk of periprosthetic femoral fracture? *Bone Joint J*. 99-B:50–59, 2017. 10.1302/0301-620X.99B1.BJJ-2016-0220.R1 28042119

[14] Konow, T., et al. Factors influencing periprosthetic femoral fracture risk. *Bone Joint J*. 103-B(4):650–658, 2021. 10.1302/0301-620X.103B4.BJJ-2020-1046.R2 33789487

[15] Ramavath, A., et al. Postoperative periprosthetic femoral fracture around total hip replacements: current concepts and clinical outcomes. *EFORT Open Rev*. 5(9):558–567, 2020. 10.1302/2058-5241.5.200003 PMC7528669 33072408

[16] Hennicke, N. S., et al. Subject specific finite element modelling of periprosthetic femoral fractures in different load cases. *J Mech Behav Biomed Mater*. 126:105059, 2022. 10.1016/j.jmbbm.2021.105059 34995835

[17] Esposito, L., et al. Towards a patient-specific estimation of intra-operative femoral fracture risk. *Comput Methods Biomech Biomed Eng*. 21(12):663–672, 2018. 10.1080/10255842.2018.1508570 30370789

[18] Petursson, T., et al. Bone mineral density and fracture risk assessment to optimize prosthesis selection in total hip replacement. *Comput Math Methods Med*. 2015:162481, 2015. 10.1155/2015/162481 PMC4568326 26417376

[19] Gargiulo, P., et al. Assessment of total hip arthroplasty by means of computed tomography 3D models and fracture risk evaluation. *Artif Org*. 37(6):567–573, 2013. 10.1111/aor.12033 23550540

[20] Hennicke, N. S., D. Kluess, and M. Sander. Influence of stem design parameters on periprosthetic femoral fractures examined by subject specific finite element analyses. *Med Eng Phys*. 119:104032, 2023. 10.1016/j.medengphy.2023.104032 37634909

[21] Seemala, V., et al., Medical vs MicroCT based finite element analysis: exploring the influence of bone heterogeneity and bone geometry, in *2024 46th Annual International Conference of the IEEE Engineering in Medicine and Biology Society (EMBC)*, 2024. p. 1–5. 10.1109/EMBC53108.2024.10782156 40038996

[22] Seemala, V., M.A. Williams, R. King, and A. Palit, Towards prediction of peri-prosthetic fractures: a comprehensive finite element model of uncemented total hip arthroplasty, in *18th International Conference on Biomedical Engineering (ICBME 2024)*, 2024: Nat’l University of Singapore (NUS), Singapore.

[23] Knowles, N. K., K. Ip, and L. M. Ferreira. The effect of material heterogeneity, element type, and down-sampling on trabecular stiffness in micro finite element models. *Ann Biomed Eng*. 47(2):615–623, 2019. 10.1007/s10439-018-02152-6 30362084

[24] Seemala, V., et al. A micro-CT based cadaveric study investigating bone density changes during hip arthroplasty surgery. *J Orthopaed Res*. 43:818–827, 2025. 10.1002/jor.26032 PMC11898157 39723646

[25] Wu, G., et al. ISB recommendation on definitions of joint coordinate system of various joints for the reporting of human joint motion—part I: ankle, hip, and spine. *J Biomech*. 35(4):543–548, 2002. 10.1016/s0021-9290(01)00222-6 11934426

[26] Palit, A., et al. Evaluating computed bony range of motion (BROM) by registering in-vitro cadaver-based functional range of motion (FROM) to a hip motion simulation. *Comput Biol Med*. 169:107799, 2024. 10.1016/j.compbiomed.2023.107799 38104517

[27] Palit, A., et al. Simulation of hip bony range of motion (BROM) corresponds to the observed functional range of motion (FROM) for pure flexion, internal rotation in deep flexion, and external rotation in minimal flexion-extension—a cadaver study. *Comput Biol Med*. 183:109270, 2024. 10.1016/j.compbiomed.2024.109270 39504777

[28] Morgan, E. F., H. H. Bayraktar, and T. M. Keaveny. Trabecular bone modulus–density relationships depend on anatomic site. *J Biomech*. 36(7):897–904, 2003. 10.1016/s0021-9290(03)00071-x 12757797

[29] Keyak, J. H., I. Y. Lee, and H. B. Skinner. Correlations between orthogonal mechanical properties and density of trabecular bone: use of different densitometric measures. *J Biomed Mater Res*. 28(11):1329–1336, 1994. 10.1002/jbm.820281111 7829563

[30] Keller, T. S. Predicting the compressive mechanical behavior of bone. *J Biomech*. 27(9):1159–1168, 1994. 10.1016/0021-9290(94)90056-6 7929465

[31] Bayraktar, H. H., et al. Comparison of the elastic and yield properties of human femoral trabecular and cortical bone tissue. *J Biomech*. 37(1):27–35, 2004. 10.1016/s0021-9290(03)00257-4 14672565

[32] Niebur, G. L., et al. High-resolution finite element models with tissue strength asymmetry accurately predict failure of trabecular bone. *Journal of Biomechanics*. 33(12):1575–1583, 2000. 10.1016/s0021-9290(00)00149-4 11006381

[33] Miles, B., et al. Subject specific finite element modeling of periprosthetic femoral fracture using element deactivation to simulate bone failure. *Med Eng Phys*. 37(6):567–573, 2015. 10.1016/j.medengphy.2015.03.012 25937546

[34] Dannaway, J., et al. AN INVESTIGATION into the FRICTIONAL PROPERTIES between BONE and VARIOUS ORTHOPEDIC IMPLANT SURFACES—IMPLANT STABILITY. *J Musculoskeletal Res*. 18(4):1–15, 2015.

[35] Biemond, J. E., R. Aquarius, N. Verdonschot, and P. Buma. Frictional and bone ingrowth properties of engineered surface topographies produced by electron beam technology. *Arch Orthop Trauma Surg*. 131(5):711–718, 2011. 10.1007/s00402-010-1218-9 PMC3078515 21161665

[36] Shirazi-Adl, A., M. Dammak, and G. Paiement. Experimental determination of friction characteristics at the trabecular bone/porous-coated metal interface in cementless implants. *J Biomed Mater Res*. 27(2):167–175, 1993. 10.1002/jbm.820270205 8436573

[37] Patton, D. M., et al. The relationship between whole bone stiffness and strength is age and sex dependent. *J Biomech*. 83:125–133, 2019. 10.1016/j.jbiomech.2018.11.030 PMC6338331 30527634

[38] Porter, D. A., et al. Modern trunnions are more flexible: a mechanical analysis of THA taper designs. *Clin Orthop Relat Res*. 472(12):3963–3970, 2014. 10.1007/s11999-014-3965-3 PMC4397791 25267272

[39] Sacher, S. E., et al. Association of total hip arthroplasty flexural rigidity with magnetic resonance imaging and histological findings. *J Arthroplasty*. 39(8):2116–2123, 2024. 10.1016/j.arth.2024.02.072 38428693

[40] Esposito, L., et al. Towards an app to estimate patient-specific perioperative femur fracture risk. *Applied Sciences*. 10(18):6409, 2020.

[41] Batz, J., et al. The influence of broach design on bone friction and osseodensification in total hip arthroplasty. *Clin Biomech*. 73:234–240, 2020. 10.1016/j.clinbiomech.2019.12.012 32062473

[42] Seemala, V., et al. Quantifying bone compaction and implant-bone contact in uncemented total hip arthroplasty through muCT and digital volume correlation: a cadaveric study. *Comput Biol Med*. 184:109474, 2024. 10.1016/j.compbiomed.2024.109474 39615236

[43] Anderson, P. M., et al. Accuracy of positioning and risk factors for malpositioning custom-made femoral stems in total hip arthroplasty-a retrospective multicenter analysis. *J Pers Med*. 13(9):1285, 2023. 10.3390/jpm13091285 PMC10532930 37763052

[44] Belzunce, M. A., J. Henckel, A. Di Laura, and A. Hart. Uncemented femoral stem orientation and position in total hip arthroplasty: a CT study. *J Orthop Res*. 38(7):1486–1496, 2020. 10.1002/jor.24627 32056292

[45] Bergmann, G., et al. Standardized loads acting in hip implants. *PLoS One*. 11(5):e0155612, 2016. 10.1371/journal.pone.0155612 PMC4873223 27195789

[46] Pancanti, A., M. Bernakiewicz, and M. Viceconti. The primary stability of a cementless stem varies between subjects as much as between activities. *J Biomech*. 36(6):777–785, 2003. 10.1016/s0021-9290(03)00011-3 12742445

[47] Kohli, N., J. C. Stoddart, and R. J. van Arkel. The limit of tolerable micromotion for implant osseointegration: a systematic review. *Sci Rep*. 11(1):10797, 2021. 10.1038/s41598-021-90142-5 PMC8144379 34031476

[48] Barnes, S. C., J. C. Clasper, A. M. J. Bull, and J. R. T. Jeffers. Micromotion and push-out evaluation of an additive manufactured implant for above-the-knee amputees. *J Orthop Res*. 37(10):2104–2111, 2019. 10.1002/jor.24389 31166039

[49] Søballe, K., et al. The effects of hydroxyapatite coating and bone allograft on fixation of loaded experimental primary and revision implants. *Acta Orthopaedica*. 74(3):239–247, 2003. 10.1080/00016470310014139 PMC4495908 12899541

[50] van Arkel, R. J., S. Ghouse, P. E. Milner, and J. R. T. Jeffers. Additive manufactured push-fit implant fixation with screw-strength pull out. *J Orthop Res*. 36(5):1508–1518, 2018. 10.1002/jor.23771 PMC6175131 29023901

[51] Duchemin, L., et al. Prediction of mechanical properties of cortical bone by quantitative computed tomography. *Med Eng Phys*. 30(3):321–328, 2008. 10.1016/j.medengphy.2007.04.008 17596993

[52] Wille, H., E. Rank, and Z. Yosibash. Prediction of the mechanical response of the femur with uncertain elastic properties. *J Biomech*. 45(7):1140–1148, 2012. 10.1016/j.jbiomech.2012.02.006 22417868

[53] Les, C. M., et al. Estimation of material properties in the equine metacarpus with use of quantitative computed tomography. *J Orthop Res*. 12(6):822–833, 1994. 10.1002/jor.1100120610 7983558

[54] Orr, T. E., G. S. Beaupré, D. R. Carter, and D. J. Schurman. Computer predictions of bone remodeling around porous-coated implants. *J Arthroplasty*. 5(3):191–200, 1990. 10.1016/s0883-5403(08)80074-5 2230816

[55] Gruen, T. A., G. M. McNeice, and H. C. Amstutz. “Modes of failure” of cemented stem-type femoral components: a radiographic analysis of loosening. *Clin Orthop Relat Res*. 141:17–27, 1979. 477100

[56] Torre, B. B., et al. Total hip arthroplasty stems and femur strain during implant insertion: a biomechanical study of single taper, double taper, and collared monolithic stems. *Orthop Surg*. 17:2445–2453, 2025. 10.1111/os.70110 PMC12318697 40619757

[57] Mathai, B., S. Dhara, and S. Gupta. Bone remodelling in implanted proximal femur using topology optimization and parameterized cellular model. *J Mech Behav Biomed Mater*. 125:104903, 2022. 10.1016/j.jmbbm.2021.104903 34717117

[58] Ghosh, R., and S. Gupta. Bone remodelling around cementless composite acetabular components: the effects of implant geometry and implant-bone interfacial conditions. *J Mech Behav Biomed Mater*. 32:257–269, 2014. 10.1016/j.jmbbm.2014.01.010 24508712

[59] Oliviero, S., M. Giorgi, and E. Dall’Ara. Validation of finite element models of the mouse tibia using digital volume correlation. *J Mech Behav Biomed Mater*. 86:172–184, 2018. 10.1016/j.jmbbm.2018.06.022 29986291

[60] Kusins, J., et al. Full-field comparisons between strains predicted by QCT-derived finite element models of the scapula and experimental strains measured by digital volume correlation. *J Biomech*. 113:110101, 2020. 10.1016/j.jbiomech.2020.110101 33171355

[61] Garavelli, C., et al. Comparing the predictions of CT-based subject-specific finite element models of human metastatic vertebrae with digital volume correlation measurements. *Biomech Model Mechanobiol*. 24(3):1017–1030, 2025. 10.1007/s10237-025-01950-x PMC12162702 40252160

[62] Kusins, J., et al. The application of digital volume correlation (DVC) to evaluate strain predictions generated by finite element models of the osteoarthritic humeral head. *Ann Biomed Eng*. 48(12):2859–2869, 2020. 10.1007/s10439-020-02549-2 PMC7723934 32572730

[63] Dall’Ara, E., D. Barber, and M. Viceconti. About the inevitable compromise between spatial resolution and accuracy of strain measurement for bone tissue: a 3D zero-strain study. *J Biomech*. 47(12):2956–2963, 2014. 10.1016/j.jbiomech.2014.07.019 25113806

